# Nucleoside macrocycles formed by intramolecular click reaction: efficient cyclization of pyrimidine nucleosides decorated with 5'-azido residues and 5-octadiynyl side chains

**DOI:** 10.3762/bjoc.14.217

**Published:** 2018-09-13

**Authors:** Jiang Liu, Peter Leonard, Sebastian L Müller, Constantin Daniliuc, Frank Seela

**Affiliations:** 1 State Key Laboratory of Oral Diseases & National Clinical Research Center for Oral Diseases & Dept. of Oral Medicine of West China Hospital of Stomatology, Sichuan University, 610041 Chengdu, Sichuan, P. R. China; 2 Laboratory of Bioorganic Chemistry and Chemical Biology, Center for Nanotechnology, Heisenbergstrasse 11, 48149 Münster, Germany; 3 Laboratorium für Organische und Bioorganische Chemie, Institut für Chemie neuer Materialien, Universität Osnabrück, Barbarastrasse 7, 49069 Osnabrück, Germany; 4 Institut für Organische Chemie, Universität Münster, Corrensstrasse 40, 48149 Münster, Germany

**Keywords:** click cyclization, conformation, macrocycles, nucleosides, X-ray

## Abstract

Copper(I)-promoted "click" cyclization in the presence of TBTA afforded nucleoside macrocycles in very high yields (≈70%) without using protecting groups. To this end, dU and dC derivatives functionalized at the 5-position of the nucleobase with octadiynyl side chains and with azido groups at the 5’-position of the sugar moieties were synthesized. The macrocycles display freely accessible Watson–Crick recognition sites. The conformation of the 16-membered macrocycle was deduced from X-ray analysis and ^1^H,^1^H-NMR coupling constants. The sugar conformation (*N* vs *S*) was different in solution as compared to the solid state.

## Introduction

The field of macrocycles was initiated by the work of Ruzicka and his structure analysis of the cyclic ketones muscone and civetone [1]. Other classical examples are cyclic peptides such as valinomycin and cyclic oligosaccharides like cyclodextrins [2–4]. The literature has been recently reviewed [5]. Also, oligonucleotides form cyclic structures commonly existing in plasmid DNA. Monomeric purine and pyrimidine nucleosides form smaller ring systems known as cyclonucleosides incorporating O, N or S-bridges within the sugar moiety or between the nucleobase and the sugar residue [6].

**Figure 1:**
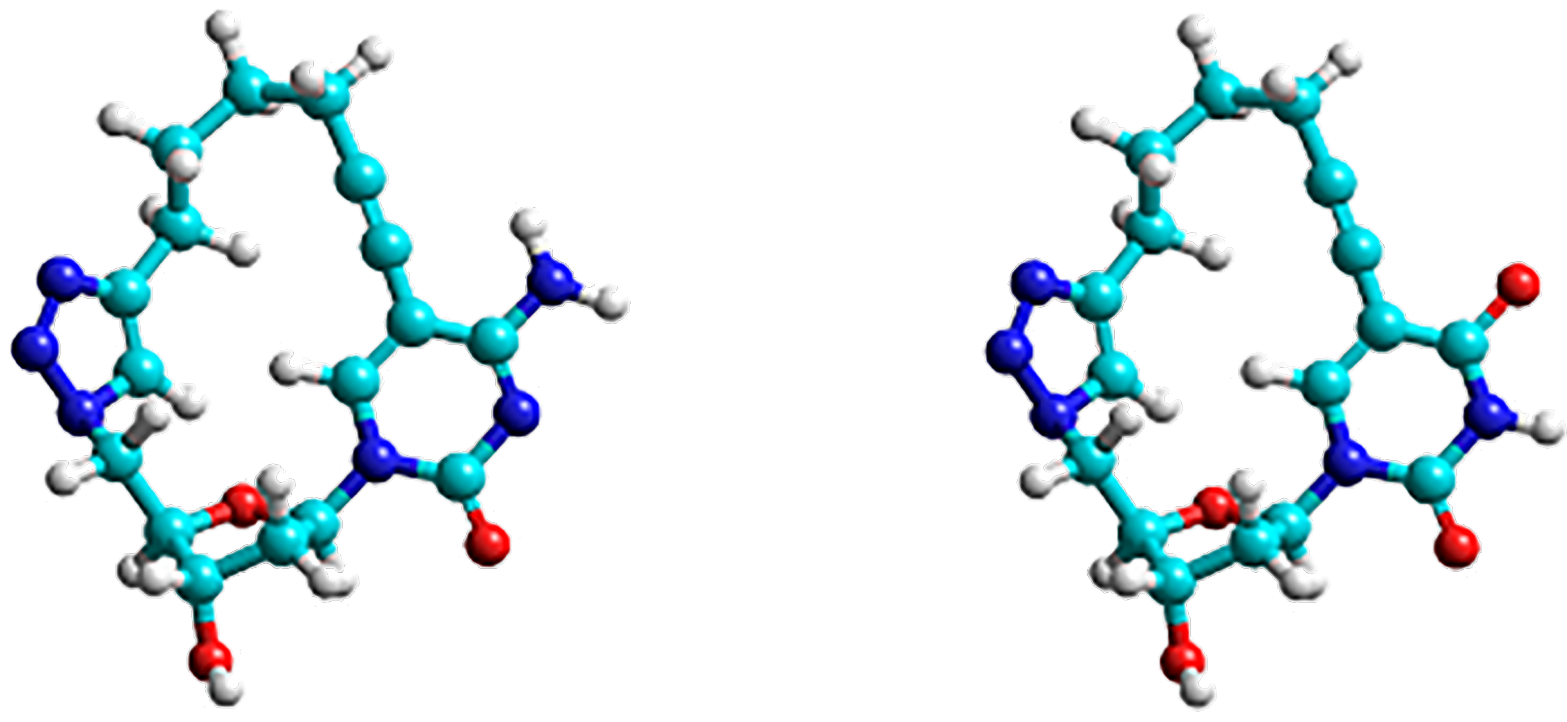
Energy-minimized models of the two macrocycles derived from dC (left) and dU (right) acquired by MM**+** simulation using Hyperchem 8.0.10; both showing the accessibility of the Watson–Crick recognition sites.

Macrocycles can be obtained by a variety of chemical reactions [7–10]. Often, several protection and deprotection steps are necessary to control the cyclization process. Preorganization of the molecules can help to make cyclization more efficient. Azide–alkyne "click" chemistry has been executed to generate cyclic peptides [11–13], cyclic oligonucleotides [14–17] and other macrocyclic systems [18–25]. DNA mimics with triazole linkages were constructed [26–27]. The click reaction was used to generate a cyclic ADP-ribose second messenger mimic [28]. Modelling studies using MM+ energy minimization showed that pyrimidine nucleosides are useful synthons for cyclic molecules when alkynyl side chains are functionalizing nucleobases in 5-position and azido substituents replace sugar 5'-hydroxy groups. Cyclic molecules (Figure 1) should be accessible when a copper(I)-azide–alkyne cycloaddition [29–31] is performed. The resulting "nucleoides" represent a new lead for a diversity of molecules. From the cyclic molecule a single crystal X-ray analysis was obtained. The sugar conformation was studied in solution and in solid state.

## Results and Discussion

**Scheme 1:**
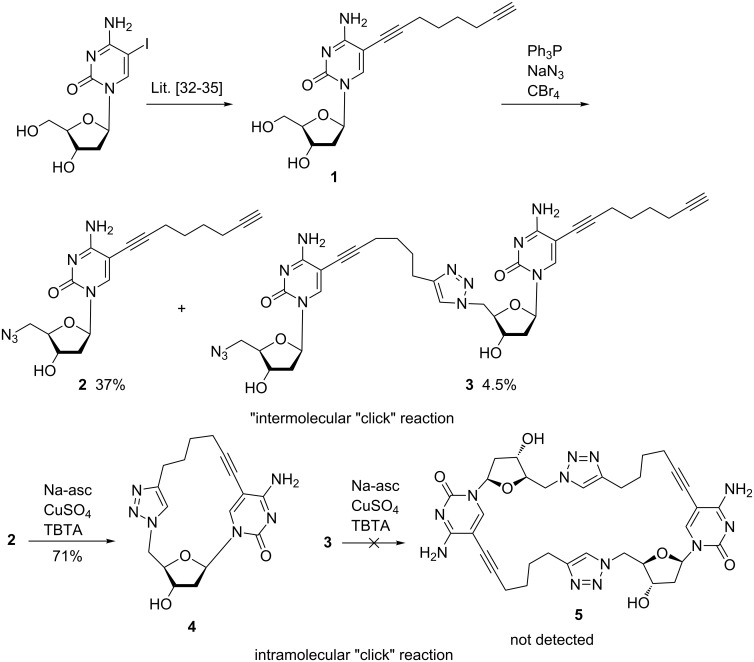
Synthesis of the 5’-azido-2’,5’-dideoxyribonucleoside **2**, the macrocycle **4** and the dimeric compounds **3** (isolated) and **5** (not detected).

The octadiynyl derivative **1** of dC [32–35] was the starting material for the synthesis of 5’-azido-2’,5’-dideoxycytidine **2**. Earlier, the nucleoside precursor **1** was used for DNA cross-linking and labelling [36]. The unprotected nucleoside **1** was treated with equimolar amounts of carbon tetrabromide and triphenylphosphine and a five-fold excess of sodium azide to obtain the azide derivative **2** (37%) together with the dimeric side product **3** (4.5%, Scheme 1) [37]. The moderate yield of the 5’azido-dC derivative results from incomplete conversion. Possibly, traces of copper used for the Sonogashira cross coupling and high substrate concentration were initiating dimerization of azide **2**. Nevertheless, an intramolecular cyclization to a macrocycle was not observed.

Next, the 5’-azido compound **2** was employed in the copper(I)-catalyzed azide–alkyne cycloaddition (CuAAC) "click" reaction [38–39] to build up macrocycle **3**. In this regard, two reaction pathways have to be considered: (i) an intramolecular “click” reaction leading to a macrocycle or (ii) an intermolecular “click” reaction forming dimeric or oligomeric compounds. For a deeper insight, the “click” reaction was executed under different reaction conditions. First, the copper(I)-promoted “click” reaction was performed on **2** in the presence of copper(II) sulfate and ascorbic acid. TLC monitoring showed that the cyclization failed.

Then, tris(benzyltriazoylmethyl)amine (TBTA) [40–42] was added as catalyst and macrocycle **4** was formed in 71% yield, which is extremely high for an intramolecular cyclization. The dimeric product **3** and the cyclic dimer **5** were not detected. Apparently, cyclization is favored over the formation of dimers due to acceleration of the reaction and the rather low concentration of starting materials (Ruggli–Ziegler dilution principle) [43].

**Scheme 2:**
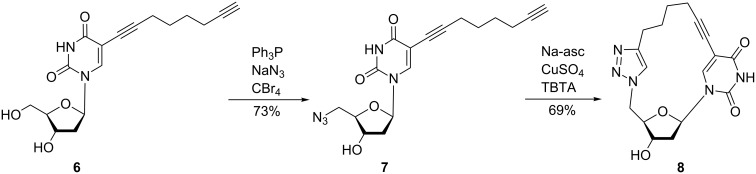
Synthesis of 5’-azido-2’,5’-dideoxyribonucleoside **7** and nucleoside macrocycle **8**.

To test the utility of the intramolecular “click” reaction, the reaction sequence performed on dC was carried out with 5-(1,7-octadiynyl)-dU (**6**) [32–33,44]. The latter was converted to 5’-azido-dU **7** using the same reaction conditions as described above (Scheme 2). By this means, compound **7** was isolated in 73% yield. Then, click cyclization was performed. In contrast to the cyclic dC derivative **4** the dU macrocycle **8** could be isolated in 46% yield even in the absence of TBTA. However, the yield of cyclization was significantly improved when TBTA was added (69%). This demonstrates the influence of the nucleobase on the intramolecular cyclization reaction.

All compounds were characterized by ESI–TOF mass spectra, ^1^H and ^13^C NMR spectroscopy as well as 2D NMR spectra (Supporting Information File 1). The NMR data gave evidence of the structural assignment of the 5’-azido compounds and the macrocycles. A strong upfield shift (≈10 ppm) for the C5’-carbon signal as well as a moderate upfield shift (3–4 ppm) of the C4’-carbon signal was observed when the 5’-OH group was replaced by an azido group or a triazole moiety (Supporting Information File 1, Table S1). Irradiation of the triazole-H of **4** and **8**, resulted in strong NOE’s at H-6, indicating that the Watson–Crick recognition sites of nucleobases are located outside of the macrocycle. The intensity of NOE’s for the CH_2_ groups decreased with increasing distance (Supporting Information File 1, Figures S21 and S35).

As the triple bonds of the macrocycles **4** and **8** are in conjugation to the nucleobases they influence the UV spectra and affect p*K*_a_ values. In both macrocycles the UV maxima are bathochromically shifted (273 to 301 nm) for **4** and 261 to 294 nm for **8** (Supporting Information File 1, Figure S1). Also, the p*K*_a_ values are affected by the cyclization. In case of dU (9.3) a decrease to 8.7 [35] for **6** and further to 8.2 for cyclic dU **8** is observed (Supporting Information File 1, Figure S2). This might go back to stacking interactions of the nucleobase and the triazole residue. A similar relationship exists in the dC series with values of 4.2 for dC and 3.0 for **1** [35]. In contrast the p*K*_a_ for cyclic dC **4** (3.2) did not further decrease.

**Figure 2:**
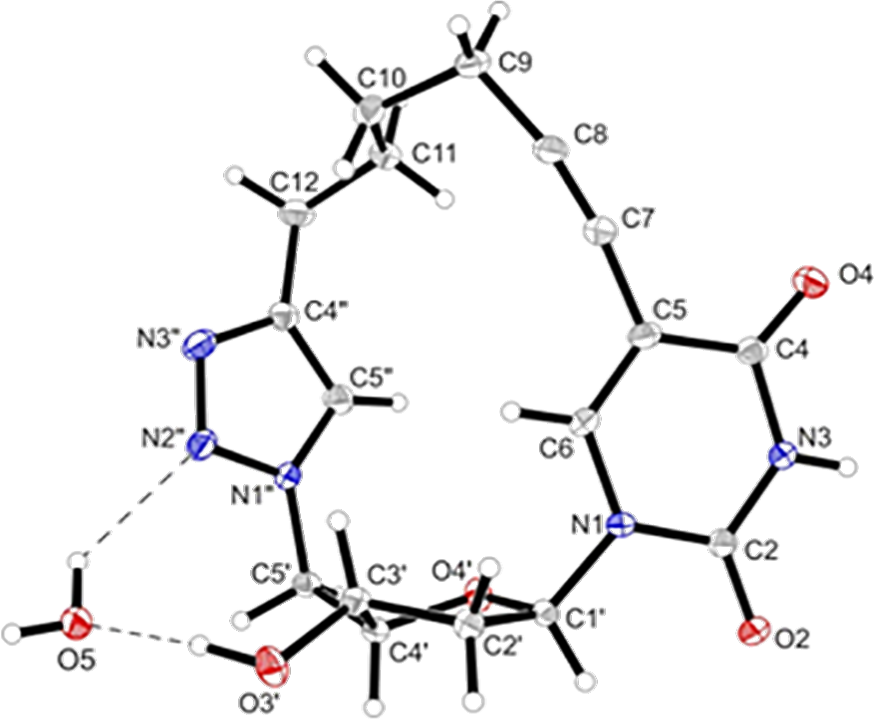
A perspective view of **8** showing the atomic numbering scheme. Displacement ellipsoids are drawn at the 50% probability level and H-atoms are shown as small spheres of arbitrary size. Hydrogen bonds are shown as dashed lines.

Next, a X-ray analysis was performed from the dU macrocycle **8**, which was crystallized from methanolic solution containing traces of water. Colorless needles with a melting point of 260–265 °C (decomp.) were obtained. The solid state structure of the macrocycle is displayed in Figure 2.

The X- ray structure of the macrocycle reflects the properties of the components with slight deviations. The glycosylic bond length (N1–C1’) of **8** is 1.459(3) Å and is in the range of 5-substituted 2’-deoxyuridines [45]. The alkynyl side chain (C5–C7–C8) and (C7–C8–C9) is slightly bend with bond angles of 172.1 (3)° and 168.6 (2)° (Figure 2). The triple bond shows a coplanar orientation to the pyrimidine ring with an inclination angle of 1.0 (4)°. The torsion angle χ [46] (−103.6° (2)) is *high*-*anti* [47]. This conformation results from restriction caused by the cyclic structure. Most nucleosides including dT (χ = 173°) [48] adopt an *anti*-conformation [49]. The conformation of the 2’-deoxyribofuranosyl moiety of **8** shows an C3’-*endo* envelope pucker (_4_E, *N*-type) in the solid state with a pseudorotational phase angle *P* = 50.2(2)° and a maximum amplitude of τ_m_ = 38.7(1)°.

**Figure 3:**
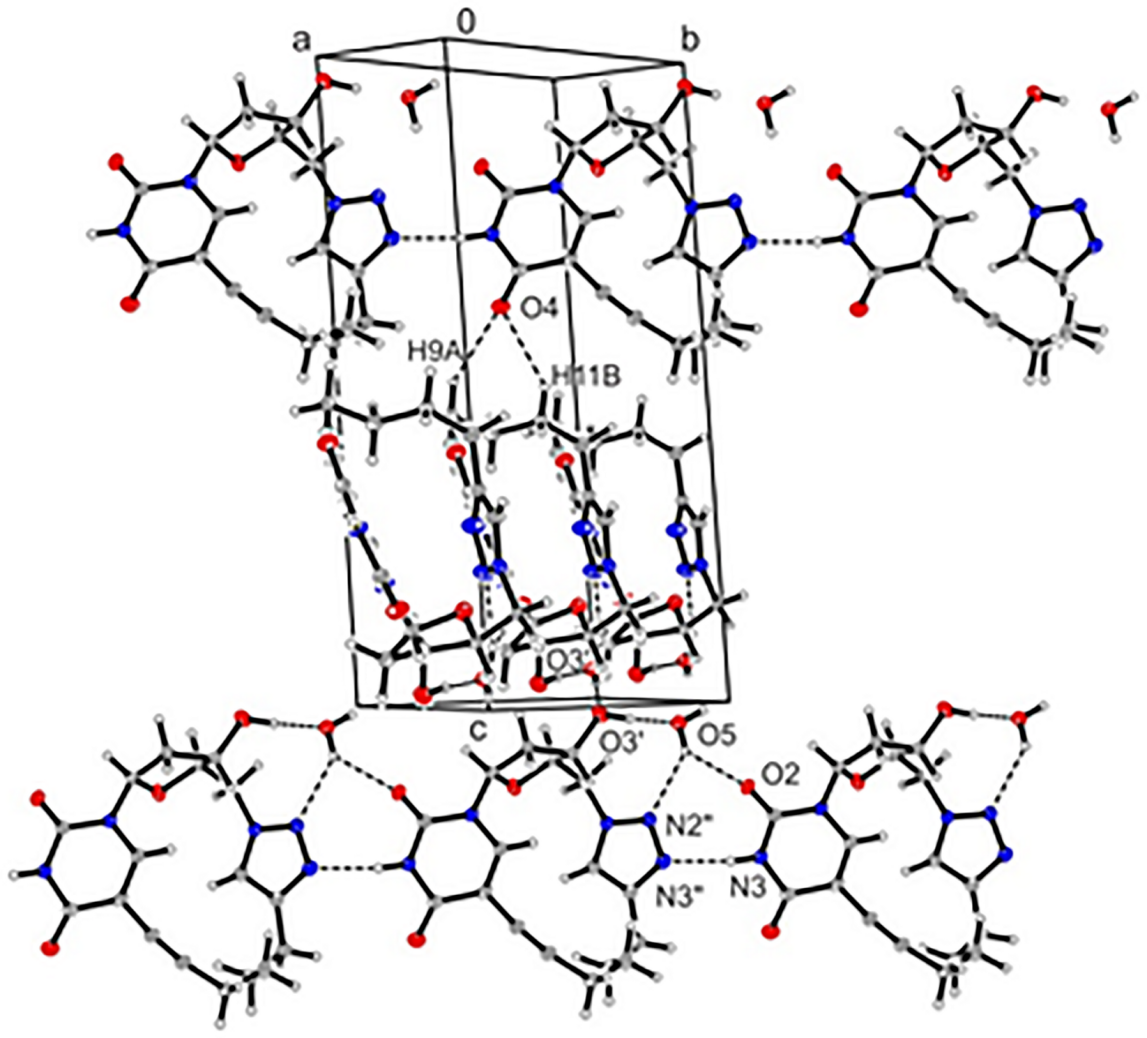
The crystal packing of **8** shows the intramolecular hydrogen-bonding network (projection parallel to the x-axis).

The extended structure forms a three-dimensional network consisting of a linear unit connected by hydrogen bonds between N3–H and the triazole N3’’ of a second molecule (Figure 3, Supporting Information File 1, Figures S3 and S4). Additionally, the molecules are bridged by water molecules connecting O2 of the base moiety and N2’’ of the triazole ring with 3’-OH of the next unit. The second layer is twisted by ≈54° to the first layer and both layers are hold together by weak hydrogen bonds between O4 and methylene groups C9 and C11. In a particular layer the triazole rings are stacked. The same is true for the base moieties.

**Figure 4:**
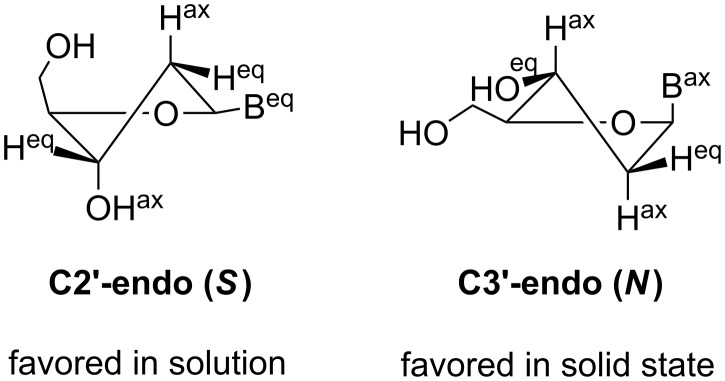
*N*- and *S*-conformation for cyclonucleoside **8**. B corresponds to nucleobase. ax: axial; eq: equatorial.

For comparison, the conformations of the 5’-azido compounds **7** and **2** as well as dC macrocycle **4** were investigated in solution. To this end, high resolution spectra (600 MHz NMR) were measured in DMSO-*d*_6_ and the population of *S* vs *N* conformers (Supporting Information File 1, Table S2) were calculated using the program PSEUROT (version 6.3) [50]. It is apparent that the 2’-deoxyribofuranosyl moiety of the open chain and the macrocyclic nucleosides favor an *S*-type sugar puckering with values of 70% *S* for azido-dU **7** and 68% for azido-dC **2**. The values for the cyclic derivatives **8** and **4** are in the same range with 72% *S*-type pucker for cyclo-dU **8** and 68% *S*-type for cyclo-dC **4**. The conformation found for the macrocycle **8** is different to that in the solid state (*N*-type, Figure 4). Apparently, the sugar residue of the macrocycle exhibits sufficient flexibility to adopt the *S*-conformation of DNA and the *N*-conformation of RNA.

## Conclusion

The synthesis of a macrocycle is often described as an intramolecular cyclization of a bifunctional precursor molecule. However, in many cases competition exists with dimerization and polymerization. Therefore, we were delighted to see that the intramolecular cyclization, utilizing dU and dC derivatives **4** and **8**, resulted in high cyclization yields (around 70%) without formation of dimeric or oligomeric molecules. The speed of the TBTA catalyzed click reaction and dilution (Ruggli–Ziegler dilution principle) [43] can be made responsible for this behavior. For the copper(I)-promoted cyclization reaction the use of the TBTA complex was essential for the cyclization of dC precursor **2** but not for dU precursor **7**. Protection of precursor molecules is not required and only four steps are necessary to convert a nucleoside in a nucleoside macrocycle. The single crystal X-ray structure confirmed the click connectivity and gave an insight to the conformation. The sugar conformation (*N* vs *S*) in solution was different to that in the solid state. The macrocycles display free accessible Watson–Crick recognition sites valuable for base pairing with nucleic acids or proteins. Since the compact nucleoside macrocycles display increased lipophilicity they have the potential to be utilized for the transmembrane delivery of nucleotides and oligonucleotides. More important, all of the macrocyle moieties and the size of the macrocycle can be altered. The system can be regarded as a new lead for further structural and functional elucidation.

## Supporting Information

File 1 Experimental procedures, analytical data, NMR spectra, conformational analysis and crystallographic data.
