# Supplementary material for: Nucleoside macrocycles formed by intramolecular click reaction: efficient cyclization of pyrimidine nucleosides decorated with 5'-azido residues and 5-octadiynyl side chains
Source: Beilstein J Org Chem. 2018 Sep 13;14:2404–10. doi: 10.3762/bjoc.14.217 (PMC6142766; doi:10.3762/bjoc.14.217)
Supplement: File 1 — Experimental procedures, analytical data, NMR spectra, conformational analysis and crystallographic data. [file Beilstein_J_Org_Chem-14-2404-s001.pdf]

Supporting Information

for

Nucleoside macrocycles formed by intramolecular click reaction: efficient cyclization of pyrimidine nucleosides decorated with 5'-azido residues and 5-octadiynyl side chains

Jiang Liu[1,2,3], Peter Leonard[2], Sebastian L. Müller[2], Constantin Daniliuc[4] and Frank Seela[*2,3]

Address: [1]State Key Laboratory of Oral Diseases & National Clinical Research Center for Oral Diseases & Dept. of Oral Medicine of West China Hospital of Stomatology, Sichuan University, 610041 Chengdu, Sichuan, P. R. China, [2]Laboratory of Bioorganic Chemistry and Chemical Biology, Center for Nanotechnology, Heisenbergstrasse 11, 48149 Münster, Germany, [3]Laboratorium für Organische und Bioorganische Chemie, Institut für Chemie neuer Materialien, Universität Osnabrück, Barbarastrasse 7, 49069 Osnabrück, Germany and [4]Institut für Organische Chemie, Universität Münster, Corrensstrasse 40, 48149 Münster, Germany

* Corresponding author

Email: Frank Seela - Frank.Seela@uni-osnabrueck.de

Experimental procedures, analytical data, NMR spectra, conformational analysis and crystallographic data

Table of contents

Experimental section ……………………………………….…..……….…….……….S3

General methods ……………………………………………..………….….……….S3

Synthetic procedures ……………….....…………………………..……….…….......S3–S7

Tables of ^{13}C NMR and ^{1}H NMR chemical shifts and coupling constants ...….......S8–S9

UV spectra of compounds 4 and 8…………………………………......…...........S10

pK_a determination by UV titration ...……....……………….………………..….….………S10

X-Ray crystal structure analysis of 8 ....S11–S21

NMR spectra ……………….……..…………………..………..……………….S22–S37

Experimental section

General methods and materials

All chemicals and solvents were of laboratory grade as obtained from commercial suppliers and were used without further purification. Thin-layer chromatography (TLC) was performed on TLC aluminium sheets covered with silica gel 60 F254 (0.2 mm). Flash column chromatography (FC): silica gel 60 (40–60 μM) at 0.4 bar. UV-spectra were recorded on a UV-spectrophotometer: λ_{max} (ε) in nm, ε in dm^3 mol^{-1} cm^{-1}. NMR spectra were measured at 599.74 MHz and 300.15 MHz for ^1H and 150.82 MHz and 75.48 MHz for ^{13}C. ^1H-^{13}C correlated (HMBC, HSQC) NMR spectra were used for the assignment of the ^{13}C signals (Table 1). The *J* values are given in Hz; δ values in ppm relative to Me_4Si as internal standard. For NMR spectra recorded in DMSO-d_6, the chemical shift of the solvent peak was set to 2.50 ppm for ^1H NMR and 39.50 ppm for ^{13}C NMR. ESI-TOF mass spectra of nucleosides were recorded on a Micro-TOF spectrometer.

Synthetic procedures

1-[5-(Azido)-2,5-dideoxy-β-D-*erythro*-pentofuranosyl]-5-octa-1,7-diynylcytosine (2)

Compound **1** (517 mg, 1.56 mmol) was dissolved in dry DMF (4 mL), then triphenylphosphine (420 mg, 1.60 mmol) and NaN_3 (507 mg, 7.80 mmol) were added. Subsequently, CBr_4 (531 mg, 1.60 mmol) was added in portions over 1 h. The resulting solution was stirred under N_2 protection overnight at r.t. CH_3OH (150 μL) was added and stirring continued for 30 min. The solvent was evaporated (oil pump, 50 °C water bath) and the remaining residue was absorbed on silica gel and applied to FC (silica gel, column 4 × 12 cm, CH_2Cl_2/CH_3OH 94:6). From the faster migrating zone compound **2** (205 mg, 37%) was isolated as a colorless solid. TLC (silica gel, CH_2Cl_2/CH_3OH 9:1): R_f 0.53. λ_{max} (CH_3OH)/nm

236 (ε/dm^3 mol^{-1} cm^{-1} 20200), 298 (9300). ^{1}H NMR (DMSO-d_6, 600 MHz) (δ, ppm): 1.52-1.64 (m, 4H, 2 × CH_2), 2.09-2.21 (m, 4H, 2 × H-2′, CH_2), 2.42 (t, *J* = 7.0 Hz, 2H, CH_2), 2.76 (t, *J* = 2.4 Hz, 1H, C≡CH), 3.59 (dd, *J* = 13.1, 4.3 Hz, 1H, H-5′), 3.62 (dd, *J* = 13.1, 6.1 Hz, 1H, H-5′), 3.86 (dt, *J* = 6.0, 4.0 Hz, 1H, H-4′), 4.14 (dq, *J* = 7.2, 3.8 Hz, 1H, H-3′), 5.37 (d, *J* = 4.2 Hz, 1H, OH-3′), 6.16 (dd, *J* = 7.3, 6.3 Hz, 1H, H-1′), 6.81 (brs, 1H, NH_{2a}), 7.74 (brs, 1H, NH_{2b}), 7.83 (s, 1H, H-6). ^{13}C NMR (DMSO-d_6, 151 MHz) (δ, ppm): 17.2, 18.5, 27.0, 27.2, 39.3, 51.7, 70.7, 71.3, 72.0, 84.3, 84.6, 85.3, 90.8, 95.4, 143.4, 153.3, 164.3. ESI-TOF *m/z* calcd for $C_{17}H_{19}N_5O_4$ [M + Na]$^+$ 379.1489, found 379.1494 (+1.3 ppm).

1-[(4-{[(5-Azido-2,5-dideoxy-β-D-*erythro*-pentofuranos-1-yl)cytosin-5-yl]hex-5-yn-1-yl}-1*H*-1,2,3-triazol-1-yl)-2,5-dideoxy-β-D-*erythro*-pentofuranosyl]-5-(octa-1,7-diyn-1-yl)cytosine (3)

Evaporation of the slower migrating zone gave compound **3** (50 mg, 4.5%) as a colorless solid. TLC (silica gel,CH_2Cl_2/MeOH, 9:1): R_f 0.2. ^{1}H NMR (DMSO-d_6, 300 MHz) (δ, ppm): 1.55-1.72 (m, 8H, 4 × CH_2), 2.09-2.23 (m, 6H, 4 × H-2’, CH_2), 2.39-2.46 (m, 4H, 2 × CH_2), 2.61 (t, *J* = 6.9 Hz, 2H, CH_2), 2.76 (t, *J* = 2.7 Hz, 1H, C≡CH), 3.58-3.61 (m, 2H, 2 × H-5’ (azido)), 3.83-3.87 (m, 1H, H-4’ (azido)), 4.01-4.11 (m, 1H, H-4’ (triazole)), 4.12-4.15 (m, 1H, H-3’ (azido)), 4.21-4.23 (m, 1H, H-3’ (triazole)), 4.60-4.62 (m, 2H, H-5’ (triazole)), 5.39 (d, *J* = 4.2 Hz, 1H, OH-3’ (azido)), 5.45 (d, *J* = 4.2 Hz, 1H, OH-3’ (triazole)), 6.09-6.20 (m, 2H, 2 × H-1’), 6.79 (br s, 2H, 2 × NH_{2a}), 7.71 (s, 1H, triazole), 7.74 (br s, 2H, 2 × NH_{2b}), 7.82 (s, 2H, 2 × H-6). ^{13}C NMR (DMSO-d_6, 75 MHz) (δ, ppm): 17.0, 18.3, 18.5, 24.2, 26.8, 26.9, 27.2, 27.9, 38.4, 38.5, 50.7, 51.4, 70.4, 70.6, 71.0, 71.6, 71.7, 84.0, 84.3, 85.0, 85.2, 90.5, 90.6, 95.27, 95.30, 122.2, 143.1, 143.4, 146.3, 153.1, 164.0, 164.1. ESI-TOF *m/z* calcd for $C_{34}H_{40}N_{12}O_6$ [M+Na]$^+$ 735.3086, found 735.3061 (-3.4 ppm).

Cyclo-5,5’-(triazol-4-yl-hex-5-ynyl)-1-(2,5-dideoxy-β-D-*erythro*-pentofuranosyl)cytosine (4)

To a solution of compound **2** (197 mg, 0.55 mmol) in THF/H_2O/*t*-BuOH (3:1:1, 4 mL), Na-ascorbate (0.55 mL, 0.55 mmol) of a freshly prepared 1 M solution in water and $CuSO_4$•TBTA complex solution (1 mL, 0.15 mmol) were added. The reaction mixture was stirred in the dark under N_2 overnight at rt. The solvent was evaporated and the residue was purified by FC (silica gel, column 3 × 15 cm, CH_2Cl_2/CH_3OH 9:1) to give compound **4** as a colorless solid (140 mg, 71%). TLC (silica gel, CH_2Cl_2/CH_3OH 9:1): R_f 0.44. λ_{max} (CH_3OH)/nm 236 (ε/dm^3 mol^{-1} cm^{-1} 16100), 301 (6300). ^{1}H NMR (DMSO-*d*$_6$, 600 MHz) (δ, ppm): 1.65 (dt, *J* = 13.6, 6.8 Hz, 1H, H-2′$_\beta$), 1.76 (p, *J* = 13.6 Hz, 2H, CH_2), 1.79-1.93 (m, 2H, CH_2), 2.15 (ddd, *J* = 13.6, 6.7, 4.7 Hz, 1H, H-2′$_\alpha$), 2.43 (dt, *J* = 17.3, 6.3 Hz, 1H, CH_2), 2.47-2.54 (m, 1H, CH_2), 2.62 (dt, *J* = 14.9, 7.1 Hz, 1H, CH_2), 2.73 (dt, *J* = 15.2, 6.9 Hz, 1H, CH_2), 4.10 (dt, *J* = 4.8, 3.5 Hz, 1H, H-4′), 4.18 (dq, *J* = 8.0, 4.3 Hz, 1H, H-3′), 4.62-4.71 (m, 2H, 2 × H-5′), 5.53 (d, *J* = 4.5 Hz, 1H, OH-3′), 6.10 (t, *J* = 6.5 Hz, 1H, H-1′), 6.70 (s, 1H, H-6), 6.89 (br s, 1H, NH_{2a}), 7.63 (br s, 1H, NH_{2b}), 7.89 (s, 1H, triazole-H). ^{13}C NMR (DMSO-*d*$_6$, 151 MHz) (δ, ppm): 17.9, 22.7, 25.5, 26.4, 40.0, 49.0, 69.3, 74.2, 83.0, 84.1, 90.6, 94.3, 123.9, 144.0, 147.6, 153.2, 163.3. ESI-TOF *m/z* calcd for $C_{17}H_{19}N_5O_4$ [M + Na]$^+$ 379.1489, found 379.1476 (-3.4 ppm).

1-[5-(Azido)-2,5-dideoxy-β-D-*erythro*-pentofuranosyl]-5-(octa-1,7-diynyl)uracil (7)

Compound **6** (196 mg, 0.59 mmol) was dissolved in dry DMF (4 mL), then triphenylphosphine (156 mg, 0.60 mmol) and NaN_3 (193 mg, 2.95 mmol) were added. Subsequently, CBr_4 (195 mg, 0.60 mmol) was added in portions over 1 h. The resulting solution was stirred under N_2 protection overnight at rt CH_3OH (150 µL) was added and stirring continued for 30 min. The solvent was evaporated (oil pump, 50 °C water bath) and

the remaining residue was absorbed on silica gel and applied to FC (silica gel, column 3 × 14 cm, CH_2Cl_2/CH_3OH 96:4) furnishing compound **7** (154 mg, 73%) as colorless solid. TLC (silica gel, CH_2Cl_2/CH_3OH 9:1): R_f 0.59. λ_{max} (CH_3OH)/nm 228 (ε/dm^3 mol^{-1} cm^{-1} 11900), 292 (12200). ^{1}H NMR (DMSO-d_6, 600 MHz) (δ, ppm): 1.53-1.61 (m, 4H, 2 × CH_2), 2.10 (ddd, J = 13.6, 6.5, 3.9 Hz, 1H, H-2′$_\alpha$), 2.19 (ddt, J = 6.7, 4.7, 2.6 Hz, 2H, CH_2), 2.31 (dt, J = 13.7, 6.9 Hz, 1H, H-2′$_\beta$), 2.38-2.40 (m, 2H, CH_2), 2.75 (t, J = 2.7 Hz, 1H, C≡CH), 3.60 (d, J = 5.1 Hz, 2H, 2 × H-5′), 3.85 (td, J = 5.1, 3.8 Hz, 1H, H-4′), 4.17 (dq, J = 6.7, 3.3 Hz, 1H, H-3′), 5.41 (d, J = 3.5 Hz, 1H, OH-3′), 6.12 (t, J = 6.8 Hz, 1H, H-1′), 7.87 (s, 1H, H-6), 11.60 (br s, 1H, NH). ^{13}C NMR (DMSO-d_6, 151 MHz) (δ, ppm): 17.2, 18.3, 27.1, 27.2, 38.5, 51.5, 70.5, 71.3, 72.8, 84.2, 84.7, 84.8, 93.1, 99.3, 142.7, 149.4, 161.6. ESI-TOF *m/z* calcd for $C_{17}H_{19}N_5O_4$ [M + Na]$^+$ 380.1329, found 380.1310 (-5.0 ppm).

Cyclo-5,5’-(triazol-4-ylhex-5-ynyl)-1-(2,5-dideoxy-β-D-*erythro*-pentofuranosyl)uracil (8)

Method 1: To a solution of **7** (180 mg, 0.50 mmol) in THF/H_2O/*t*-BuOH (3:1:1, 7 mL), sodium ascorbate (0.5 mL, 0.5 mmol) of a freshly prepared 1 M solution in water and copper(II) sulphate pentahydrate 7.5% in water (0.33 mL, 0.1 mmol) were added. The reaction mixture was stirred vigorously in the dark at room temperature overnight. For completion of the reaction a second portion of sodium ascorbate (0.5mL, 0.5 mmol) and copper(II) sulphate pentahydrate 7.5% in water (0.41 mL, 0.125 mmol) were added. The reaction mixture was stirred overnight at rt. Then, the solvent was evaporated, and the residue was purified by FC (silica gel, column 3 × 10 cm, CH_2Cl_2/MeOH, 92:8) to give **8** as a colorless solid (82 mg, 46%). A small amount of the material described above was crystallized from MeOH furnishing colorless needles. M.P.: 260-265°C (decomp.). TLC (silica gel, CH_2Cl_2/CH_3OH 9:1): R_f 0.51. λ_{max} (CH_3OH)/nm 226 (ε/dm^3 mol^{-1} cm^{-1} 15400), 294 (10100). ^{1}H NMR (DMSO-d_6, 600 MHz) (δ, ppm): 1.70-1.91 (m, 5H, 2 × CH_2, H-2′$_\beta$), 2.12 (ddd, J =

13.6, 6.7, 4.4 Hz, 1H, H-2′$_{\alpha}$), 2.40 (dt, *J* = 17.4, 6.4 Hz, 1H, CH_2), 2.47 (dt, *J* = 17.3, 6.1 Hz, 1H, CH_2), 2.60 (dt, *J* = 15.0, 7.2 Hz, 1H, CH_2), 2.73 (dt, *J* = 15.2, 6.9 Hz, 1H, CH_2), 4.10 (dt, *J* = 4.9, 3.6 Hz, 1H, H-4′), 4.17 (dq, *J* = 8.4, 4.5 Hz, 1H, H-3′), 4.64-4.70 (m, 2H, 2 × H-5′), 5.56 (d, *J* = 4.4 Hz, 1H, OH-3′), 6.08 (t, *J* = 6.7 Hz, 1H, H-1′), 6.71 (s, 1H, H-6), 7.86 (s, 1H, triazole-H), 11.57 (s, 1H, NH). ^{13}C NMR (DMSO-*d*$_6$, 151 MHz) (δ, ppm): 17.5, 22.7, 25.4, 26.4, 39.2, 49.0, 69.2, 74.8, 83.1, 83.4, 92.6, 99.1, 123.7, 142.6, 147.6, 149.2, 161.1. ESI-TOF *m/z* calcd for $C_{17}H_{19}N_5O_4$ $[M + Na]^+$ 380.1329, found 380.1322 (-1.8 ppm).

Method 2: To a solution of compound **7** (180 mg, 0.5 mmol) in THF/H_2O/*t*-BuOH (3:1:1, 4 mL), Na-ascorbate (0.5 mL, 0.5 mmol) of a freshly prepared 1 M solution in water and $CuSO_4$•TBTA complex solution (860 µL, 0.13 mmol) prepared by mixing copper(II) sulphate pentahydrate 7.5% in water (1 mL) and *tris*-(benzyltriazolylmethyl)amine (TBTA) (159 mg, 0.3 mmol) in THF/H_2O/*t*-BuOH (3:1:1, 1 mL). The reaction mixture was stirred in the dark under N_2 overnight at r.t. The solvent was evaporated and the residue was purified by FC (silica gel, column 3 × 12 cm, CH_2Cl_2/CH_3OH 92:8) to give compound **8** as a colorless solid (125 mg, 69%). Analytical data were identical to those described above.

Table S1 ^{13}C NMR chemical shifts of pyrimidine derivatives[a,b]

	C2[c]	C4	C5	C6[c]	C≡C	CH_2	C1'	C2'	C3'	C4'	C5'	triazole
**1** [35]	153.9	164.3	95.4	143.5	90.3, 84.2, 72.1, 71.2	17.2, 18.5, 27.1, 27.2	84.6	*d*	70.2	87.5	60.9	
**2**	153.3	164.3	95.4	143.4	90.8, 84.3, 72.0, 71.3	17.2, 18.5, 27.0, 27.2	85.3	39.3	70.7	84.6	51.7	
**3**	153.1	164.0, 164.1	95.27, 95.30	143.1, 143.4	90.6, 90.5, 71.7, 71.6, 71.0	17.0, 18.3, 18.5, 24.2, 26.8, 26.9, 27.2, 27.9	85.0 85.2	38.4 38.5	70.4 70.6	84.0 84.3	50.7 51.4	122.2, 146.3
**4**	153.2	163.3	94.3	144.0	90.6, 74.2	26.4, 25.5, 22.7, 17.9	84.1	40.0	69.3	83.0	49.0	123.9, 147.6
**6** [44]	149.4	161.7	98.9	142.6	92.9, 84.2, 72.9, 71.3	17.2, 18.3, 27.1, 27.2	84.6	*d*	70.2	87.5	60.9	
**7**	149.4	161.6	99.3	142.7	93.1, 84.2, 72.8, 71.3	17.2, 18.3, 27.1, 27.2	84.8	38.5	70.5	84.7	51.5	
**8**	149.2	161.1	99.1	142.6	92.6, 74.8	17.5, 22.7, 25.4, 26.4	83.4	39.2	69.2	83.1	49.0	123.7, 147.6

[a]Measured in DMSO-d_6 at 298 K. [b]Pyrimidine numbering. [c]Tentative. [d]Superimposed by DMSO.

Table S2 ^{1}H NMR chemical shift and proton-proton vicinal and geminal coupling constants and conformation[a,b]

	Chemical shift/ppm												Coupling constant/Hz [*J*(HH)]									Conformation	
	H-1’	H-2’	H-2”	H-3’	H-4’	H-5’	H-5”	H-6	C≡CH	Triazole H	NH_2	NH	1’2’	1’2”	2’2”	2’,3’	2”,3’	3’,4’	4’5’	4’5”	5’5”	%*N*	%*S*
**2**	6.16 (dd)	2.09-2.21 (m)	2.09 -2.21 (m)	4.14 (dq)	3.86 (dt)	3.62 (dd)	3.59 (dd)	7.83 (s)	2.76 (t)	-	6.81 (s), 7.74 (s)	-	7.1	6.3	-13.7	7.0	3.8	3.9	6.1	4.2	-13.1	32	68
**4**	6.10 (t)	1.65 (dt)	2.15 (ddd)	4.18 (dq)	4.10 (dt)	4.62-4.71 (m)	4.62-4.71 (m)	6.70 (s)	-	7.89 (s)	6.89 (s), 7.63 (s)	-	6.7	6.6	-13.6	7.4	4.5	4.6	3.5	3.5	-15.1	37	63
**7**	6.12 (t)	2.31 (dt)	2.10 (ddd)	4.17 (dq)	3.85 (td)	3.60 (d)	3.60 (d)	7.87 (s)	2.75 (t)	-	-	11.60 (s)	6.9	6.7	-13.7	6.8	3.6	3.6	5.1	5.1	-	30	70
**8**	6.08 (t)	1.70-1.91 (m)	2.12 (ddd)	4.17 (dq)	4.10 (dt)	4.64-4.70 (m)	4.64-4.70 (m)	6.71 (s)	-	7.86 (s)	-	11.57 (s)	6.8	6.8	-13.6	8.4	4.5	4.7	3.6	3.6	-15.1	28	72

[a]Measured in DMSO-d_6 at 298 K; rms < 0.4 Hz. H-2’= H-2′$_{\beta}$; H-2”= H-2′$_{\alpha}$. [b] For PSEUROT calculations the coupling constants ^{3}J(H1’-H2’), ^{3}J(H1’-H2’’), ^{3}J(H2’-H3’), ^{3}J(H2’’-H3’) and ^{3}J(H3’-H4’) were used.

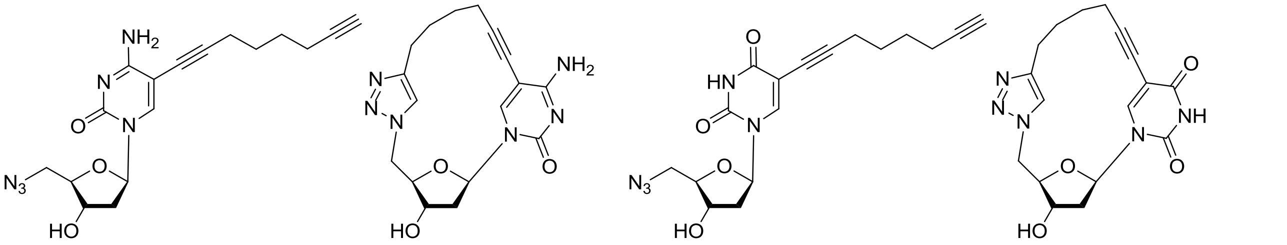

2 **4** **7** **8**

UV spectra of compounds 4 and 8

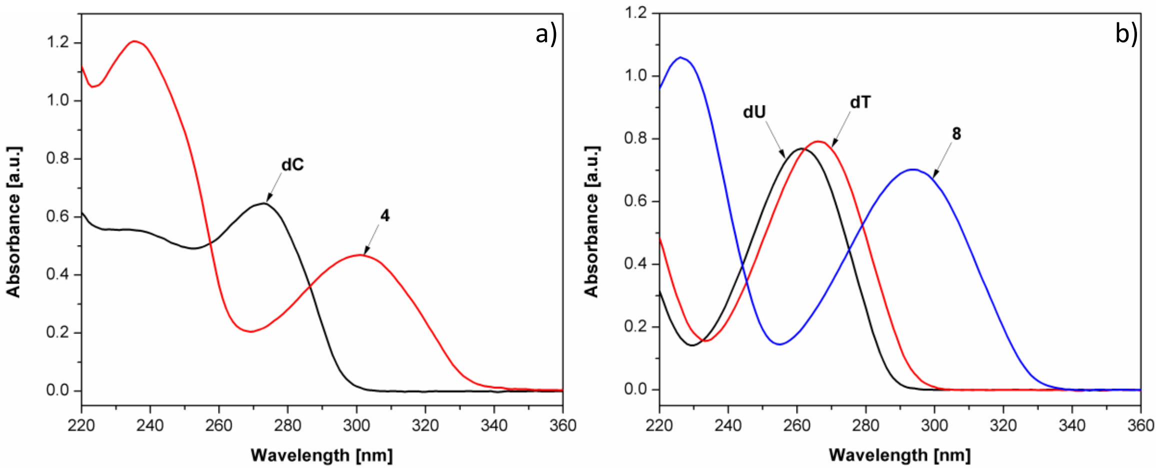

Figure S1: (a) UV spectra of the macrocyclic dC (**4**) and the nucleoside dC measured in methanol; (b) UV spectra of the macrocyclic dU (**8**) and the nucleosides dU and dT measured in methanol.

p*K*$_a$ Determination by UV titration

Macrocycles **4** and **8** were dissolved in 0.1 M sodium phosphate buffer, pH 4.4. An aqueous NaOH solution (4 M) and concentrated phosphorus acid were used to adjust the pH value of the buffer. At defined pH values, the UV absorbance of nucleosides was measured (Figure S2).

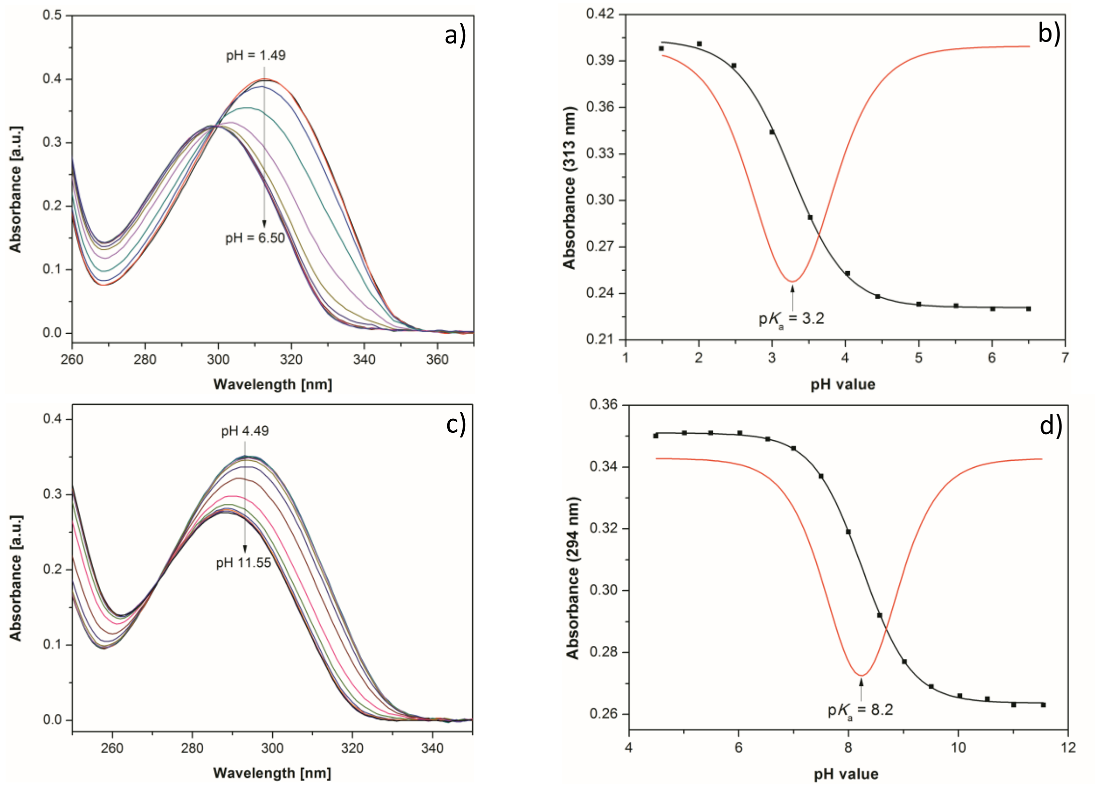

Figure S2 (a) UV spectroscopic changes of macrocycle **4** at different pH values measured in 0.1 M sodium phosphate buffer; (b) pK_a Value of **4** determined by UV titration in 0.1 M sodium phosphate buffer (c) UV spectroscopic changes of macrocycle **8** at different pH values measured in 0.1 M sodium phosphate buffer; (d) pK_a Value of **8** determined by UV titration in 0.1 M sodium phosphate buffer.

X-ray crystal structure analysis of 8

Data sets for compound **3** were collected with a D8 Venture Dual Source 100 CMOS diffractometer. Programs used: data collection: APEX3 V2016.1-0 (Bruker AXS Inc., **2016**); cell refinement: SAINT V8.37A (Bruker AXS Inc., **2015**); data reduction: SAINT V8.37A (Bruker AXS Inc., **2015**); absorption correction, SADABS V2014/7 (Bruker AXS Inc., **2014**); structure solution SHELXT-2015 (Sheldrick, **2015**); structure refinement SHELXL-2015

(Sheldrick, **2015**). *R*-values are given for observed reflections, and wR^2 values are given for all reflections. A colorless needle-like specimen of $C_{17}H_{19}N_5O_4 \cdot H_2O$, approximate dimensions 0.046 mm × 0.064 mm × 0.383 mm, was used for the X-ray crystallographic analysis. The X-ray intensity data were measured. A total of 1135 frames were collected. The total exposure time was 22.16 hours. The frames were integrated with the Bruker SAINT software package (*APEX3* (**2016**), *SAINT* (**2015**) and *SADABS* (**2014**), Bruker AXS Inc., Madison, Wisconsin, USA) using a wide-frame algorithm. The integration of the data using a monoclinic unit cell yielded a total of 10906 reflections to a maximum θ angle of 68.29° (0.83 Å resolution), of which 3205 were independent (average redundancy 3.403, completeness = 100.0%, R_{int} = 3.38%, R_{sig} = 3.24%) and 3095 (96.57%) were greater than $2\sigma(F^2)$. The final cell constants of *a* = 5.4157(2) Å, *b* = 8.9681(2) Å, *c* = 18.1307(5) Å, β = 95.2990(10)°, volume = 876.82(4) Å^3, are based upon the refinement of the XYZ-centroids of 9047 reflections above 20 σ(I) with 4.894° < 2θ<136.2°. Data were corrected for absorption effects using the multi-scan method (SADABS).The ratio of minimum to maximum apparent transmission was 0.872. The calculated minimum and maximum transmission coefficients (based on crystal size) are 0.7250 and 0.9600. The structure was solved and refined using the Bruker SHELXTL Software Package (*SHELX* software: Sheldrick, G. M. *ActaCryst.,* 2015, *A71*, 3–8), using the space group $P2_1$, with Z = 2 for the formula unit, $C_{17}H_{19}N_5O_4 \cdot H_2O$. The final anisotropic full-matrix least-squares refinement on F^2 with 260 variables converged at R1 = 2.84%, for the observed data and wR2 = 6.83% for all data. The goodness-of-fit was 1.062. The largest peak in the final difference electron density synthesis was 0.140 e^-/Å^3 and the largest hole was −0.238 e^-/Å^3 with an RMS deviation of 0.044 e^-/Å^3. On the basis of the final model, the calculated density was 1.422 g/cm^3 and F(000), 396 e^-. Flack parameter was refined to −0.02(9). CCDC Nr.: 1847724.

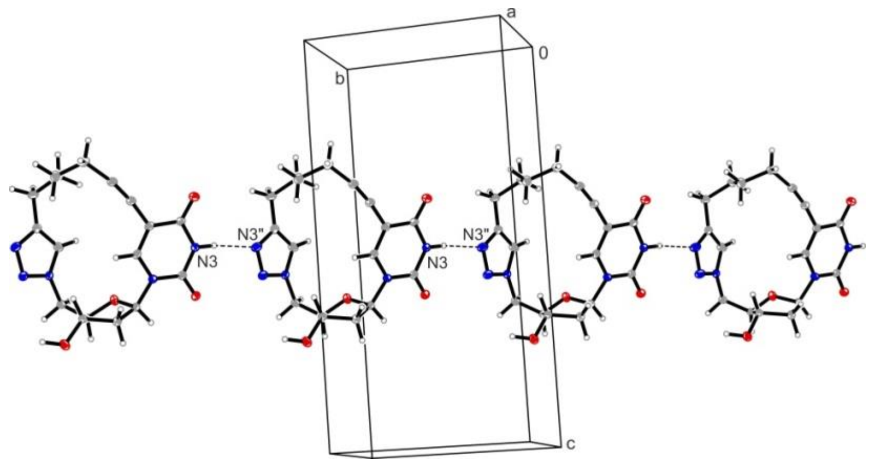

Figure S3. Linear chains perpendicular to ab-diagonal involving N-H...N hydrogen bonds in compound **8**.

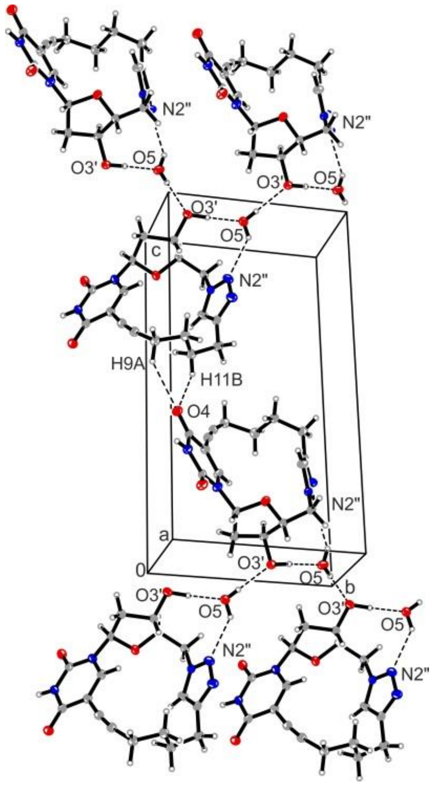

Figure S4. Excerpt of the packing diagram of **8** presenting the interaction mode through the formation of hydrogen bonds (O-H...O, O-H...N, N-H...N and C-H...O).

Table S3. Data collection details for **8.**

Axis	dx/mm	2θ/°	ω/°	ϕ/°	χ/°	Width/°	Frames	Time/s	Wavelength/Å	Voltage/kV	Current/mA	Temperature/K
Omega	33.997	104.27	108.98	-135.00	-44.94	1.50	62	80.00	1.54184	50	1.0	102
Phi	33.997	104.27	14.94	-195.75	23.00	1.50	133	80.00	1.54184	50	1.0	102
Phi	33.997	89.27	92.06	0.00	-44.44	1.50	240	80.00	1.54184	50	1.0	102
Omega	33.997	-2.99	-100.60	-90.00	44.94	1.50	62	20.00	1.54184	50	1.0	102
Omega	33.997	104.27	-3.44	-45.00	62.19	1.50	72	80.00	1.54184	50	1.0	102
Omega	33.997	104.27	108.98	45.00	-44.94	1.50	62	80.00	1.54184	50	1.0	102
Phi	33.997	-0.18	357.21	-19.50	44.44	1.50	122	20.00	1.54184	50	1.0	102
Omega	33.997	104.27	108.98	-180.00	-44.94	1.50	62	80.00	1.54184	50	1.0	102
Omega	33.997	104.27	108.98	90.00	-44.94	1.50	62	80.00	1.54184	50	1.0	102
Omega	33.997	104.27	108.98	-90.00	-44.94	1.50	62	80.00	1.54184	50	1.0	102
Omega	33.997	104.27	-3.44	0.00	62.19	1.50	72	80.00	1.54184	50	1.0	102
Omega	33.997	104.27	108.98	135.00	-44.94	1.50	62	80.00	1.54184	50	1.0	102
Omega	33.997	104.27	108.98	0.00	-44.94	1.50	62	80.00	1.54184	50	1.0	102

Table S4. Sample and crystal data for 8.

**CCDC-Nr.**	1847724	
**Chemical formula**	$C_{17}H_{21}N_5O_5$	
**Formula weight**	375.39 g/mol	
**Temperature**	102(2) K	
**Wavelength**	1.54178 Å	
**Crystal size**	0.046 x 0.064 x 0.383 mm	
**Crystal habit**	colorless needle	
**Crystal system**	monoclinic	
**Space group**	P 1 21 1	
**Unit cell dimensions**	a = 5.4157(2) Å	α = 90°
	b = 8.9681(2) Å	β = 95.2990(10)°
	c = 18.1307(5) Å	γ = 90°
**Volume**	876.82(4) $Å^3$	
**Z**	2	
**Density (calculated)**	1.422 g/cm^3	
**Absorption coefficient**	0.896 mm^{-1}	
**F(000)**	396	

Table S5. Data collection and structure refinement for **8.**

**Theta range for data collection**	2.45 to 68.29°
**Index ranges**	-6<=h<=6, -10<=k<=10, -21<=l<=21
**Reflections collected**	10906
**Independent reflections**	3205 [R(int) = 0.0338]
**Coverage of independent reflections**	100.0%
**Absorption correction**	multi-scan
**Max. and min. transmission**	0.9600 and 0.7250
**Structure solution technique**	direct methods
**Structure solution program**	SHELXL-2014/7 (Sheldrick, 2014)
**Refinement method**	Full-matrix least-squares on F^2
**Refinement program**	SHELXL-2014/7 (Sheldrick, 2014)
**Function minimized**	$\Sigma w(F_o^2 - F_c^2)^2$
**Data / restraints / parameters**	3205 / 1 / 260
**Goodness-of-fit on F^2**	1.062
**Final R indices**	3095 data; I>2σ(I) R1 = 0.0284, wR2 = 0.0676
	all data R1 = 0.0299, wR2 = 0.0683
**Weighting scheme**	$w=1/[\sigma^2(F_o^2)+(0.0347P)^2+0.1480P]$ where $P=(F_o^2+2F_c^2)/3$
**Absolute structure parameter**	-0.02(9)
**Largest diff. peak and hole**	0.140 and -0.238 eÅ^{-3}
**R.M.S. deviation from mean**	0.044 eÅ^{-3}

Table S6. Atomic coordinates and equivalent isotropic atomic displacement parameters (Å^2) for **8.**

U(eq) is defined as one third of the trace of the orthogonalized U_{ij} tensor.

	**x/a**	**y/b**	**z/c**	**U(eq)**
N1	0.3355(3)	0.2858(2)	0.31252(9)	0.0155(4)
N3	0.1442(4)	0.1050(2)	0.23550(10)	0.0152(4)
N1"	0.4847(3)	0.7990(2)	0.28911(9)	0.0129(4)
N2"	0.7057(4)	0.8649(2)	0.29613(10)	0.0215(4)
N3"	0.7838(4)	0.8705(2)	0.22916(10)	0.0215(4)
O2	0.0004(3)	0.15611(18)	0.34663(8)	0.0193(4)
O4	0.2431(3)	0.06690(18)	0.11804(8)	0.0204(4)
O4'	0.2484(3)	0.52596(17)	0.35104(9)	0.0165(3)
O3'	0.7834(3)	0.63436(19)	0.47179(8)	0.0192(3)
O5	0.8773(3)	0.93221(19)	0.45330(10)	0.0211(4)
C2	0.1490(4)	0.1804(2)	0.30115(12)	0.0150(4)
C4	0.2858(4)	0.1334(3)	0.17651(11)	0.0150(4)
C5	0.4776(4)	0.2457(3)	0.19263(12)	0.0161(5)
C6	0.4916(4)	0.3166(3)	0.25848(12)	0.0168(5)
C1'	0.3393(4)	0.3829(2)	0.37716(11)	0.0161(5)
C2'	0.5963(4)	0.4096(3)	0.41827(12)	0.0173(5)
C3'	0.6459(4)	0.5754(3)	0.40781(11)	0.0142(4)
C4'	0.3823(4)	0.6379(3)	0.39478(11)	0.0143(4)
C5'	0.3467(4)	0.7859(3)	0.35480(11)	0.0145(4)
C5"	0.4192(4)	0.7623(3)	0.21786(12)	0.0190(5)
C4"	0.6124(4)	0.8093(2)	0.17929(12)	0.0154(4)
C12	0.6407(4)	0.8116(3)	0.09793(12)	0.0204(5)
C11	0.6718(4)	0.6578(3)	0.06248(12)	0.0184(5)
C10	0.9281(4)	0.5910(3)	0.08016(13)	0.0193(5)
C9	0.9445(4)	0.4278(3)	0.05533(12)	0.0182(5)
C8	0.7828(4)	0.3377(3)	0.09805(12)	0.0173(5)
C7	0.6419(4)	0.2873(3)	0.13850(12)	0.0173(5)

Table S7. Bond lengths (Å) for **8.**

N1-C6	1.380(3)	N1-C2	1.385(3)
N1-C1'	1.459(3)	N3-C2	1.367(3)
N3-C4	1.396(3)	N3-H3	0.88(4)
N1"-N2"	1.330(3)	N1"-C5"	1.349(3)
N1"-C5'	1.468(3)	N2"-N3"	1.323(3)
N3"-C4"	1.352(3)	O2-C2	1.224(3)
O4-C4	1.220(3)	O4'-C4'	1.434(3)
O4'-C1'	1.438(3)	O3'-C3'	1.421(3)
O3'-H3A	0.99(5)	O5-H5A	0.80(4)
O5-H5B	0.87(4)	C4-C5	1.457(3)
C5-C6	1.349(3)	C5-C7	1.434(3)
C6-H6	0.95	C1'-C2'	1.536(3)
C1'-H1	1.0	C2'-C3'	1.526(3)
C2'-H2A	0.99	C2'-H2B	0.99
C3'-C4'	1.531(3)	C3'-H3B	1.0
C4'-C5'	1.516(3)	C4'-H4A	1.0
C5'-H5C	0.99	C5'-H5D	0.99
C5"-C4"	1.377(3)	C5"-H5E	0.95
C4"-C12	1.497(3)	C12-C11	1.537(3)
C12-H12A	0.99	C12-H12B	0.99
C11-C10	1.519(3)	C11-H11A	0.99
C11-H11B	0.99	C10-C9	1.536(3)
C10-H10A	0.99	C10-H10B	0.99
C9-C8	1.465(3)	C9-H9A	0.99
C9-H9B	0.99	C8-C7	1.195(3)

Table S8. Bond angles (°) for **8**.

C6-N1-C2	121.13(18)	C6-N1-C1'	119.15(18)
C2-N1-C1'	118.89(17)	C2-N3-C4	127.5(2)
C2-N3-H3	115.5(19)	C4-N3-H3	116.8(19)
N2"-N1"-C5"	110.76(17)	N2"-N1"-C5'	118.61(17)
C5"-N1"-C5'	130.49(18)	N3"-N2"-N1"	106.89(17)
N2"-N3"-C4"	109.88(18)	C4'-O4'-C1'	107.57(17)
C3'-O3'-H3A	109.(3)	H5A-O5-H5B	104.(4)
O2-C2-N3	122.5(2)	O2-C2-N1	122.49(19)
N3-C2-N1	114.99(18)	O4-C4-N3	120.2(2)
O4-C4-C5	125.94(19)	N3-C4-C5	113.86(18)
C6-C5-C7	119.7(2)	C6-C5-C4	118.95(19)
C7-C5-C4	121.24(19)	C5-C6-N1	123.1(2)
C5-C6-H6	118.4	N1-C6-H6	118.4
O4'-C1'-N1	106.80(17)	O4'-C1'-C2'	106.76(17)
N1-C1'-C2'	115.31(18)	O4'-C1'-H1	109.3
N1-C1'-H1	109.3	C2'-C1'-H1	109.3
C3'-C2'-C1'	104.67(18)	C3'-C2'-H2A	110.8
C1'-C2'-H2A	110.8	C3'-C2'-H2B	110.8
C1'-C2'-H2B	110.8	H2A-C2'-H2B	108.9
O3'-C3'-C2'	110.24(18)	O3'-C3'-C4'	113.61(18)
C2'-C3'-C4'	101.74(18)	O3'-C3'-H3B	110.3
C2'-C3'-H3B	110.3	C4'-C3'-H3B	110.3
O4'-C4'-C5'	108.23(17)	O4'-C4'-C3'	104.23(18)
C5'-C4'-C3'	118.01(18)	O4'-C4'-H4A	108.7
C5'-C4'-H4A	108.7	C3'-C4'-H4A	108.7
N1"-C5'-C4'	113.99(18)	N1"-C5'-H5C	108.8
C4'-C5'-H5C	108.8	N1"-C5'-H5D	108.8
C4'-C5'-H5D	108.8	H5C-C5'-H5D	107.6
N1"-C5"-C4"	105.5(2)	N1"-C5"-H5E	127.3
C4"-C5"-H5E	127.3	N3"-C4"-C5"	106.97(18)
N3"-C4"-C12	121.8(2)	C5"-C4"-C12	131.0(2)
C4"-C12-C11	115.14(19)	C4"-C12-H12A	108.5
C11-C12-H12A	108.5	C4"-C12-H12B	108.5
C11-C12-H12B	108.5	H12A-C12-H12B	107.5
C10-C11-C12	113.48(19)	C10-C11-H11A	108.9
C12-C11-H11A	108.9	C10-C11-H11B	108.9
C12-C11-H11B	108.9	H11A-C11-H11B	107.7
C11-C10-C9	112.97(19)	C11-C10-H10A	109.0
C9-C10-H10A	109.0	C11-C10-H10B	109.0
C9-C10-H10B	109.0	H10A-C10-H10B	107.8
C8-C9-C10	108.47(18)	C8-C9-H9A	110.0
C10-C9-H9A	110.0	C8-C9-H9B	110.0
C10-C9-H9B	110.0	H9A-C9-H9B	108.4
C7-C8-C9	168.6(2)	C8-C7-C5	172.1(3)

Table S9. Torsion angles (°) for **8**.

C5"-N1"-N2"-N3"	-0.1(3)	C5'-N1"-N2"-N3"	-176.38(19)
N1"-N2"-N3"-C4"	0.4(3)	C4-N3-C2-O2	172.7(2)
C4-N3-C2-N1	-7.6(3)	C6-N1-C2-O2	-176.3(2)
C1'-N1-C2-O2	-6.9(3)	C6-N1-C2-N3	4.0(3)
C1'-N1-C2-N3	173.47(19)	C2-N3-C4-O4	-172.1(2)
C2-N3-C4-C5	7.6(3)	O4-C4-C5-C6	175.7(2)
N3-C4-C5-C6	-4.1(3)	O4-C4-C5-C7	-1.0(4)
N3-C4-C5-C7	179.3(2)	C7-C5-C6-N1	178.1(2)
C4-C5-C6-N1	1.4(3)	C2-N1-C6-C5	-1.4(3)
C1'-N1-C6-C5	-170.8(2)	C4'-O4'-C1'-N1	-144.58(16)
C4'-O4'-C1'-C2'	-20.7(2)	C6-N1-C1'-O4'	66.0(2)
C2-N1-C1'-O4'	-103.6(2)	C6-N1-C1'-C2'	-52.4(3)
C2-N1-C1'-C2'	138.0(2)	O4'-C1'-C2'-C3'	-3.9(2)
N1-C1'-C2'-C3'	114.5(2)	C1'-C2'-C3'-O3'	145.67(17)
C1'-C2'-C3'-C4'	24.8(2)	C1'-O4'-C4'-C5'	163.51(16)
C1'-O4'-C4'-C3'	37.1(2)	O3'-C3'-C4'-O4'	-156.33(17)
C2'-C3'-C4'-O4'	-37.87(19)	O3'-C3'-C4'-C5'	83.6(2)
C2'-C3'-C4'-C5'	-157.90(18)	N2"-N1"-C5'-C4'	-95.4(2)
C5"-N1"-C5'-C4'	89.2(3)	O4'-C4'-C5'-N1"	-73.2(2)
C3'-C4'-C5'-N1"	44.7(3)	N2"-N1"-C5"-C4"	-0.2(3)
C5'-N1"-C5"-C4"	175.5(2)	N2"-N3"-C4"-C5"	-0.5(3)
N2"-N3"-C4"-C12	174.8(2)	N1"-C5"-C4"-N3"	0.4(3)
N1"-C5"-C4"-C12	-174.3(2)	N3"-C4"-C12-C11	115.0(2)
C5"-C4"-C12-C11	-70.9(3)	C4"-C12-C11-C10	-75.5(2)
C12-C11-C10-C9	170.64(18)	C11-C10-C9-C8	-64.6(2)
C10-C9-C8-C7	0.2(13)		

Table S10. Anisotropic atomic displacement parameters (Å^2) for **8**.

The anisotropic atomic displacement factor exponent takes the form: $-2\pi^2[h^2 a^{*2} U_{11} + ... + 2 h k a^* b^* U_{12}]$

	**U_{11}**	**U_{22}**	**U_{33}**	**U_{23}**	**U_{13}**	**U_{12}**
N1	0.0184(9)	0.0161(9)	0.0130(9)	-0.0025(7)	0.0069(7)	-0.0038(8)
N3	0.0176(9)	0.0148(10)	0.0137(8)	-0.0007(7)	0.0042(7)	-0.0035(8)
N1"	0.0132(8)	0.0121(8)	0.0136(9)	0.0006(7)	0.0020(7)	-0.0014(7)
N2"	0.0215(10)	0.0282(11)	0.0147(9)	0.0004(8)	0.0016(8)	-0.0123(9)
N3"	0.0198(10)	0.0293(11)	0.0159(9)	0.0020(8)	0.0036(7)	-0.0079(9)
O2	0.0222(8)	0.0200(8)	0.0168(7)	-0.0013(6)	0.0082(6)	-0.0073(6)
O4	0.0268(9)	0.0198(8)	0.0150(8)	-0.0022(7)	0.0041(7)	-0.0006(7)
O4'	0.0159(7)	0.0139(8)	0.0195(8)	-0.0023(6)	0.0006(6)	-0.0019(6)
O3'	0.0217(8)	0.0167(8)	0.0180(7)	-0.0017(6)	-0.0047(6)	-0.0004(7)
O5	0.0275(9)	0.0200(9)	0.0155(8)	0.0004(7)	0.0011(7)	-0.0082(8)
C2	0.0181(11)	0.0120(11)	0.0151(10)	0.0007(8)	0.0035(8)	0.0004(8)
C4	0.0168(11)	0.0135(10)	0.0154(10)	0.0008(8)	0.0045(8)	0.0027(9)
C5	0.0169(11)	0.0172(11)	0.0150(10)	0.0017(8)	0.0061(8)	0.0013(9)
C6	0.0180(10)	0.0159(11)	0.0174(10)	0.0000(8)	0.0066(8)	-0.0038(9)
C1'	0.0225(12)	0.0120(10)	0.0147(10)	-0.0031(8)	0.0071(8)	-0.0025(9)
C2'	0.0202(11)	0.0153(11)	0.0166(10)	0.0003(8)	0.0031(8)	-0.0012(9)
C3'	0.0151(10)	0.0158(11)	0.0120(9)	-0.0010(8)	0.0019(8)	-0.0015(8)
C4'	0.0154(10)	0.0146(10)	0.0135(9)	-0.0023(8)	0.0046(8)	-0.0014(9)
C5'	0.0133(10)	0.0162(11)	0.0145(10)	-0.0024(8)	0.0042(8)	0.0002(8)
C5"	0.0165(11)	0.0237(13)	0.0166(10)	-0.0031(9)	0.0009(8)	-0.0030(9)
C4"	0.0171(10)	0.0129(11)	0.0163(10)	0.0003(8)	0.0022(8)	0.0023(9)
C12	0.0262(11)	0.0200(12)	0.0159(11)	0.0004(9)	0.0063(9)	0.0019(10)
C11	0.0197(11)	0.0198(12)	0.0160(10)	-0.0021(9)	0.0036(8)	-0.0001(9)
C10	0.0155(11)	0.0196(12)	0.0234(11)	-0.0005(9)	0.0052(9)	-0.0027(9)
C9	0.0169(11)	0.0204(13)	0.0182(11)	0.0014(9)	0.0064(8)	0.0010(10)
C8	0.0196(11)	0.0176(12)	0.0153(10)	-0.0012(8)	0.0050(9)	0.0014(9)
C7	0.0199(11)	0.0173(11)	0.0153(10)	-0.0011(9)	0.0044(9)	-0.0004(9)

Table S11. Hydrogen atomic coordinates and isotropic atomic displacement parameters (Å^2) for **8**.

	x/a	y/b	z/c	U(eq)
H3	0.030(6)	0.034(4)	0.2288(16)	0.029(8)
H3A	0.824(9)	0.739(6)	0.463(3)	0.084(15)
H5A	0.976(7)	0.989(5)	0.474(2)	0.044(10)
H5B	0.866(7)	0.961(4)	0.407(2)	0.047(10)
H6	0.6148	0.3912	0.2681	0.02
H1	0.2257	0.3416	0.4125	0.019
H2A	0.5960	0.3849	0.4715	0.021
H2B	0.7236	0.3483	0.3967	0.021
H3B	0.7374	0.5914	0.3630	0.017
H4A	0.3109	0.6456	0.4436	0.017
H5C	0.1679	0.7998	0.3396	0.017
H5D	0.3996	0.8671	0.3897	0.017
H5E	0.2711	0.7141	0.1984	0.023
H12A	0.7870	0.8733	0.0895	0.024
H12B	0.4932	0.8607	0.0723	0.024
H11A	0.5470	0.5887	0.0799	0.022
H11B	0.6383	0.6671	0.0081	0.022
H10A	0.9733	0.5969	0.1343	0.023
H10B	1.0501	0.6509	0.0555	0.023
H9A	0.8914	0.4193	0.0018	0.022
H9B	1.1177	0.3921	0.0640	0.022

Table S12. Hydrogen bond distances (Å) and angles (°) for **8**.

	Donor-H	Acceptor-H	Donor-Acceptor	Angle
N3-H3···N3"	0.88(4)	1.99(4)	2.864(3)	172.(3)
O3'-H3A···O5	0.99(5)	1.76(5)	2.745(2)	174.(4)
O5-H5A···O3'	0.80(4)	2.03(4)	2.837(2)	178.(4)
O5-H5B···N2"	0.87(4)	2.29(4)	2.976(3)	136.(3)
O5-H5B···O2	0.87(4)	2.23(4)	2.908(2)	135.(3)
C2'-H2B···O2	0.99	2.51	3.489(3)	170.3
C5'-H5C···N2"	0.99	2.62	3.606(3)	174.1

NMR spectra

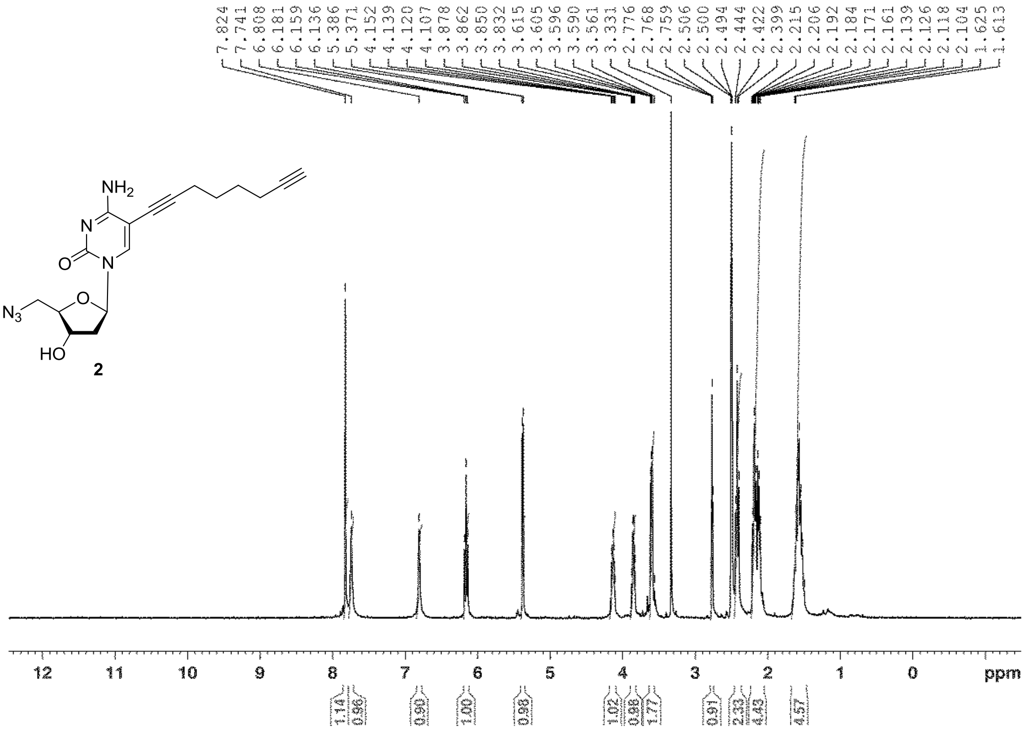

Figure S5. ^{1}H NMR spectrum of compound **2**.

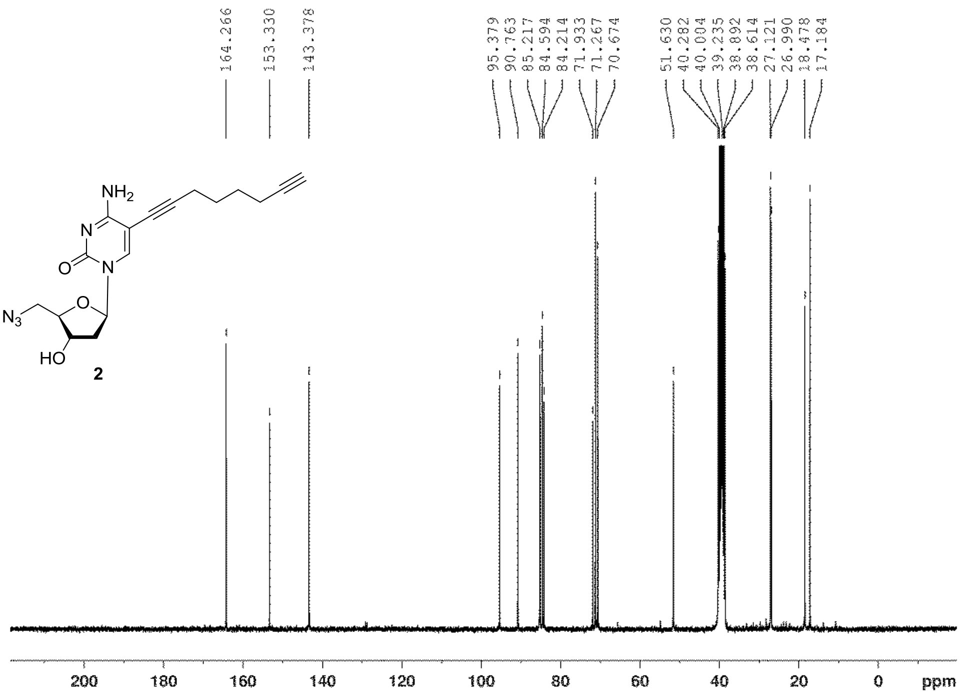

Figure S6. ^{13}C NMR spectrum of compound **2**.

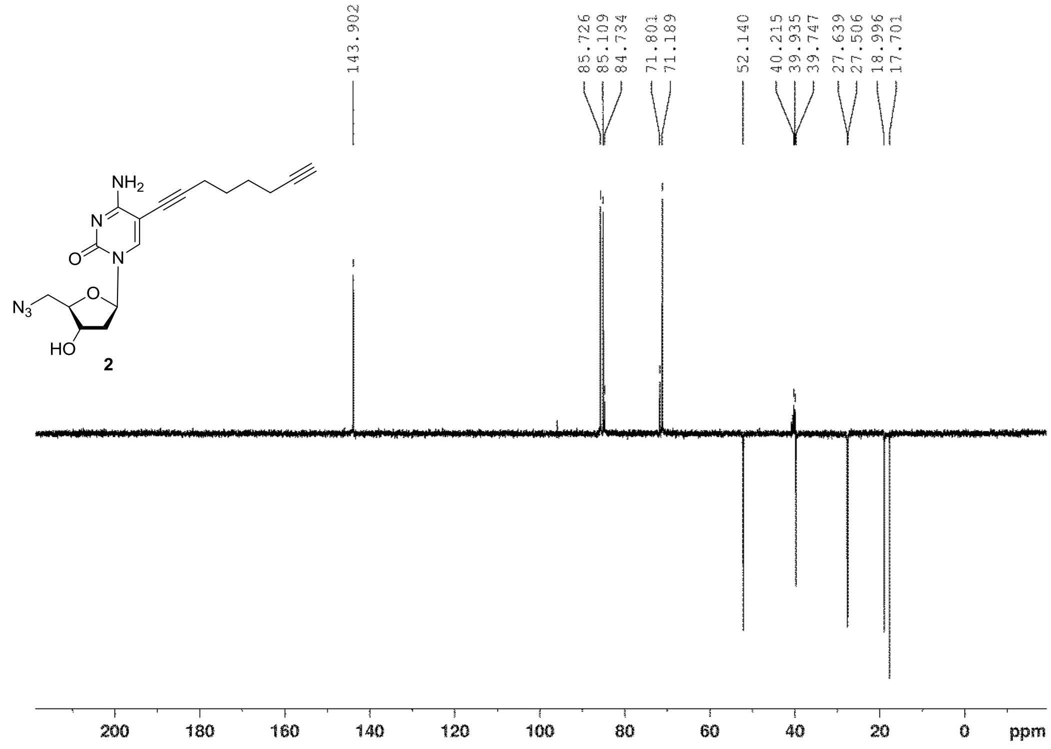

Figure S7. Dept 135 NMR spectrum of compound **2**.

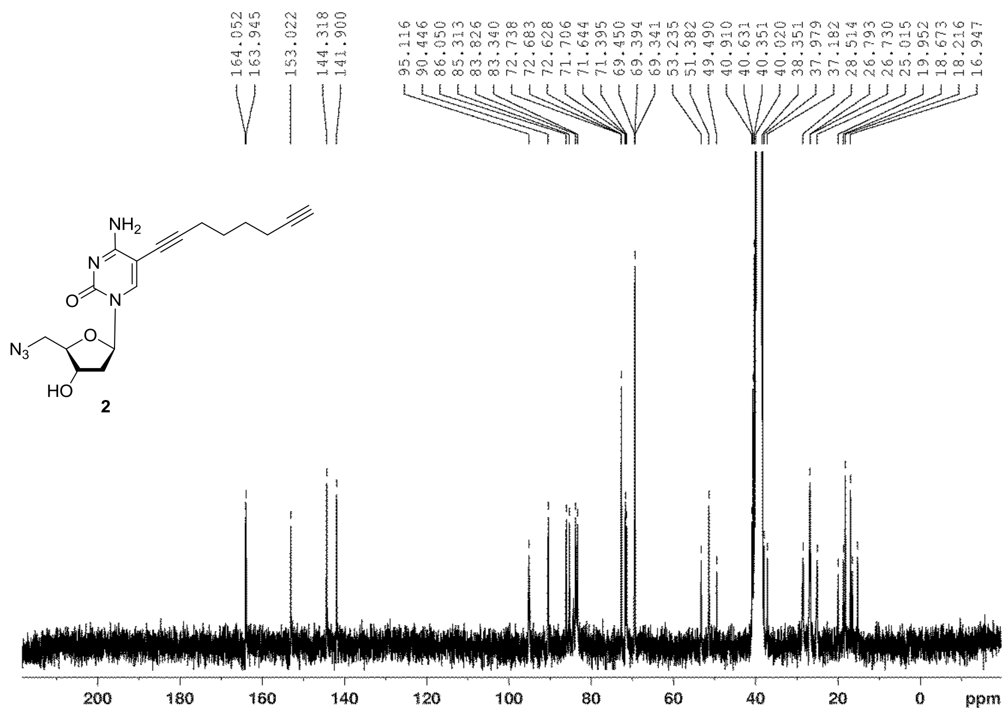

Figure S8. ^{1}H-^{13}C gated-decoupled spectrum of compound **2**.

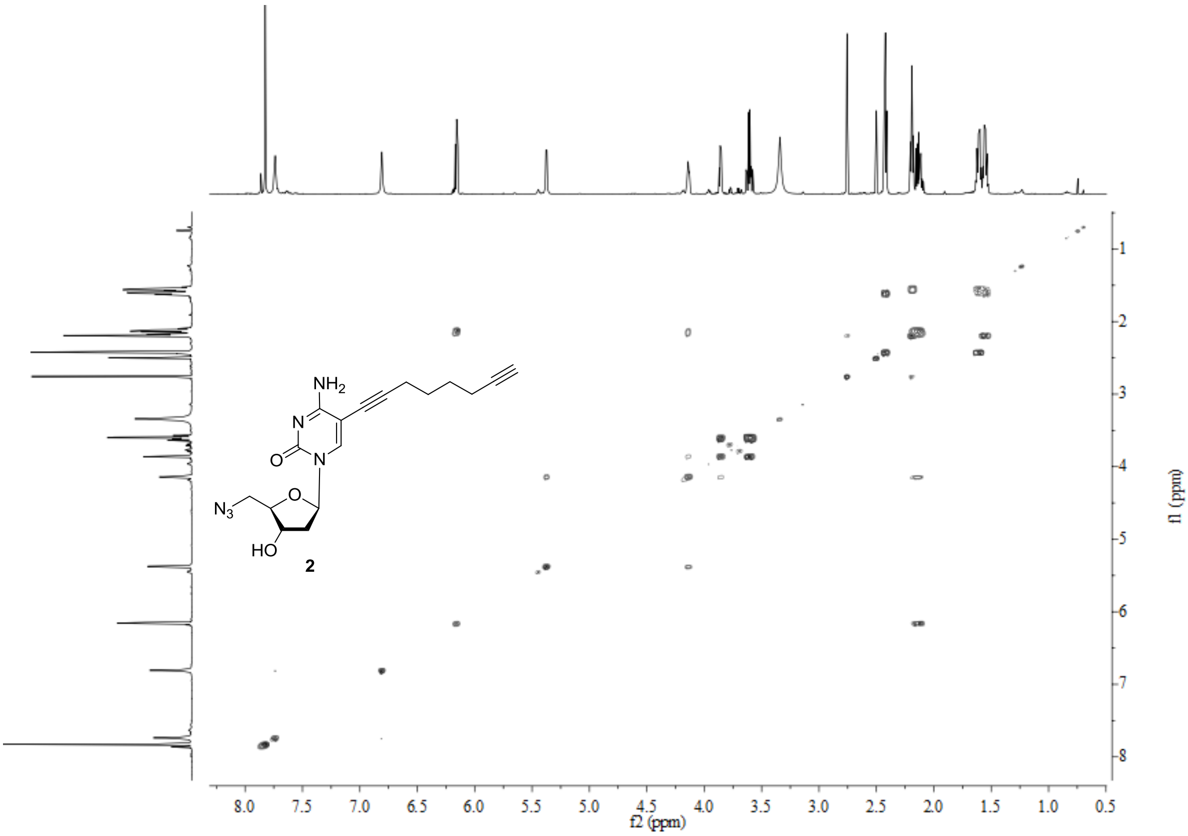

Figure S9. COSY spectrum of compound **2**.

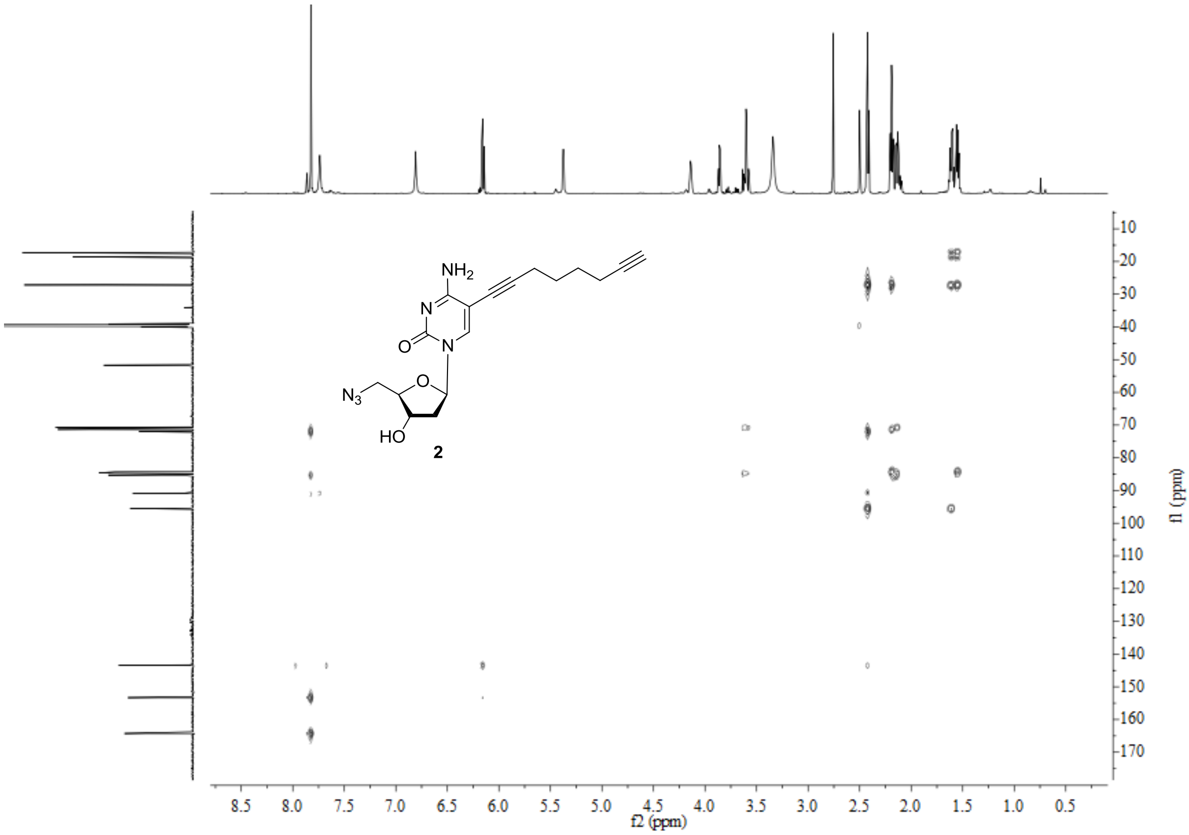

Figure S10. HMBC spectrum of compound **2**.

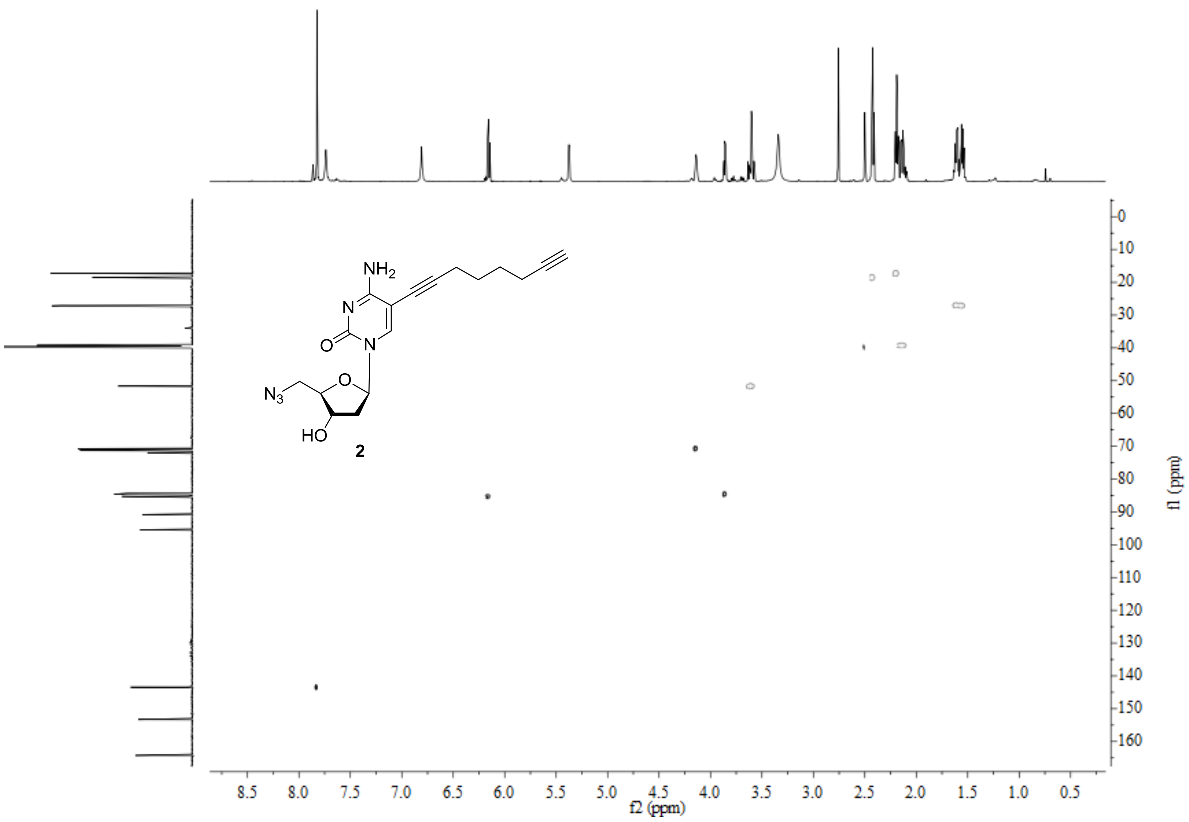

Figure S11. HSQC spectrum of compound **2**.

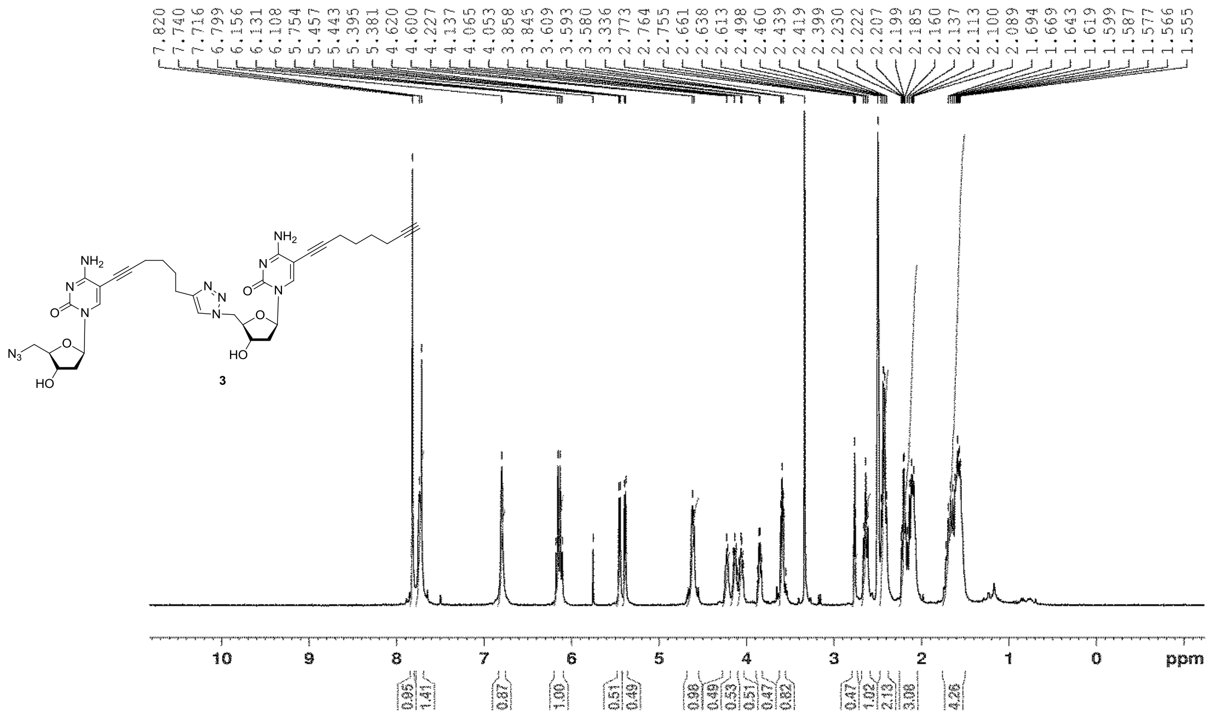

Figure S12. ^{1}H NMR spectrum of compound **3**.

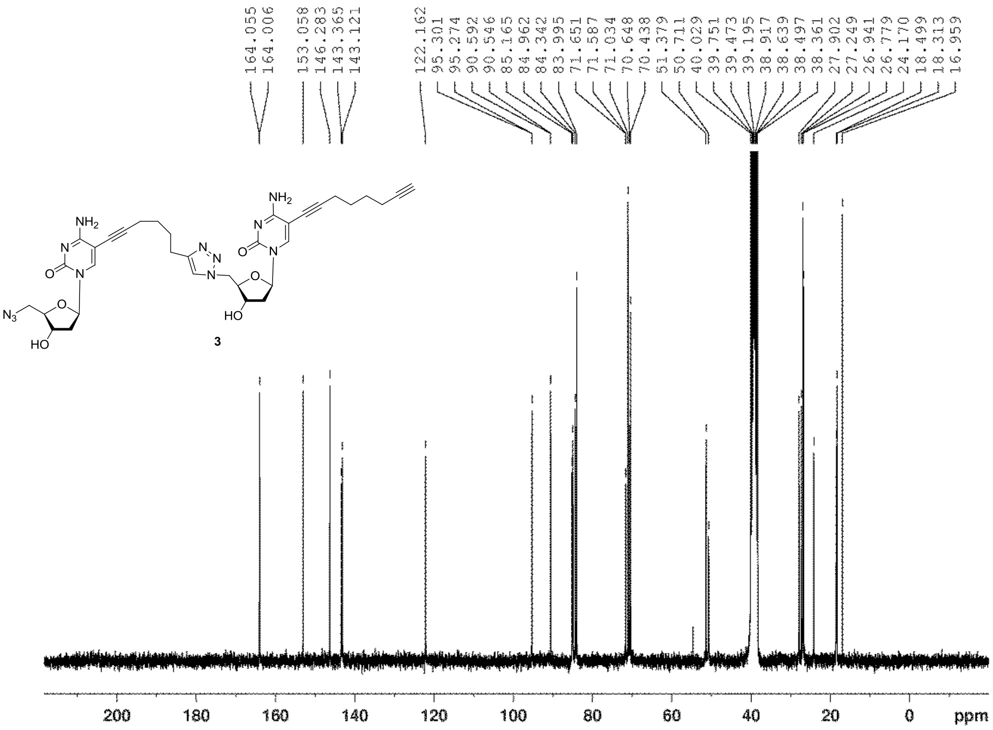

Figure S13. ^{13}C NMR spectrum of compound **3**.

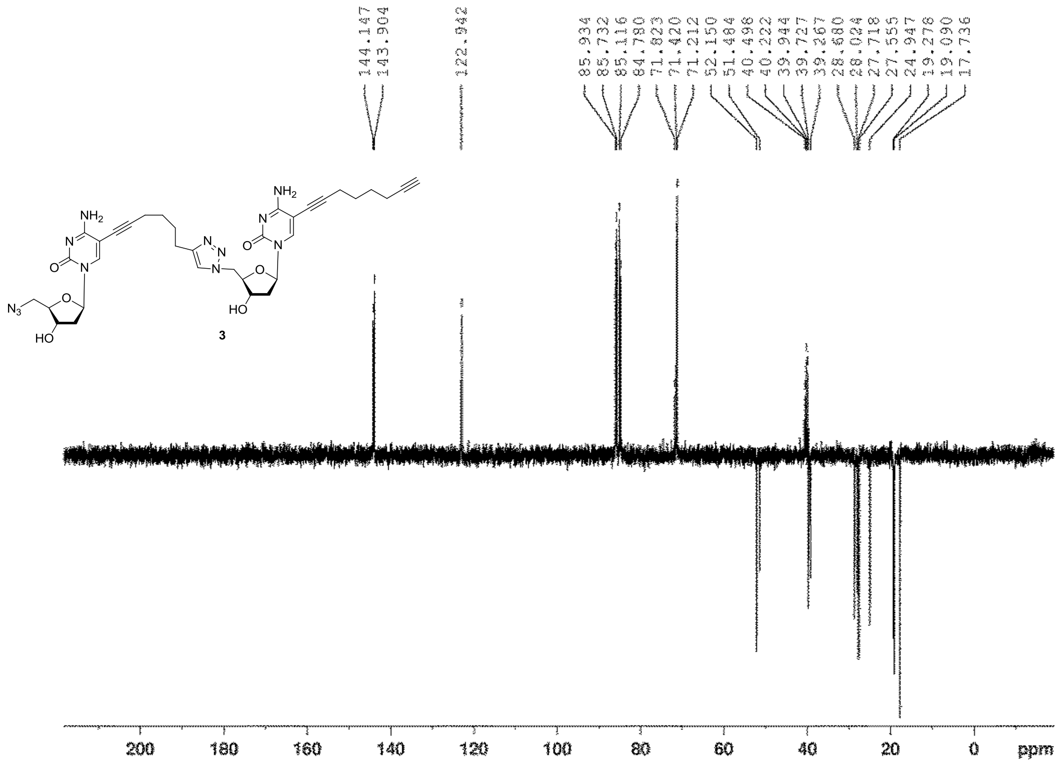

Figure S14. Dept 135 NMR spectrum of compound **3**.

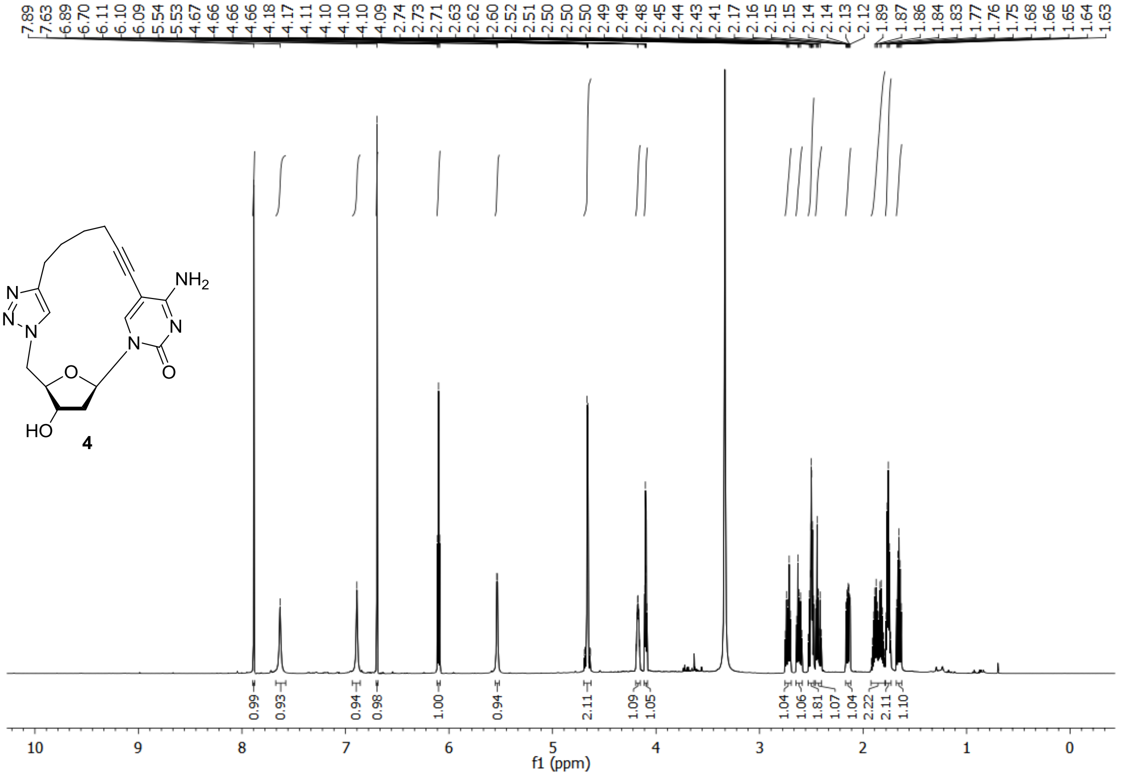

Figure S15. ^{1}H NMR spectrum of compound **4**.

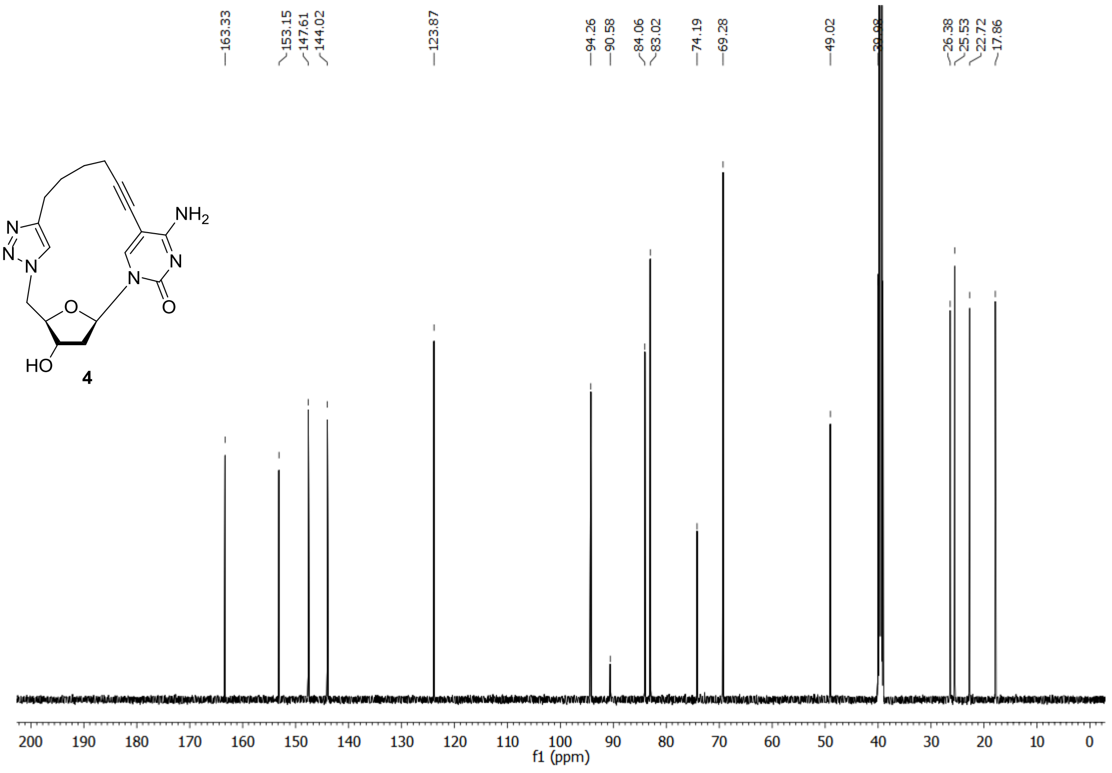

Figure S16. ^{13}C NMR spectrum of compound **4**.

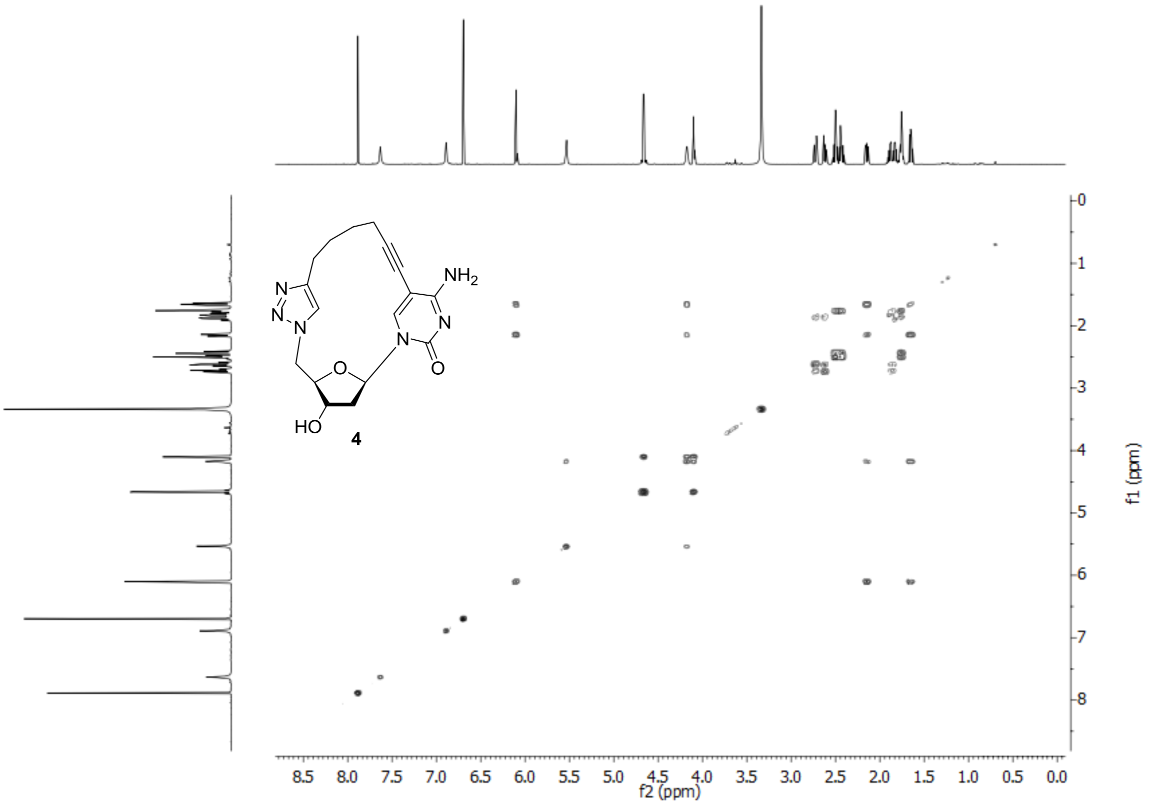

Figure S17. COSY spectrum of compound **4**.

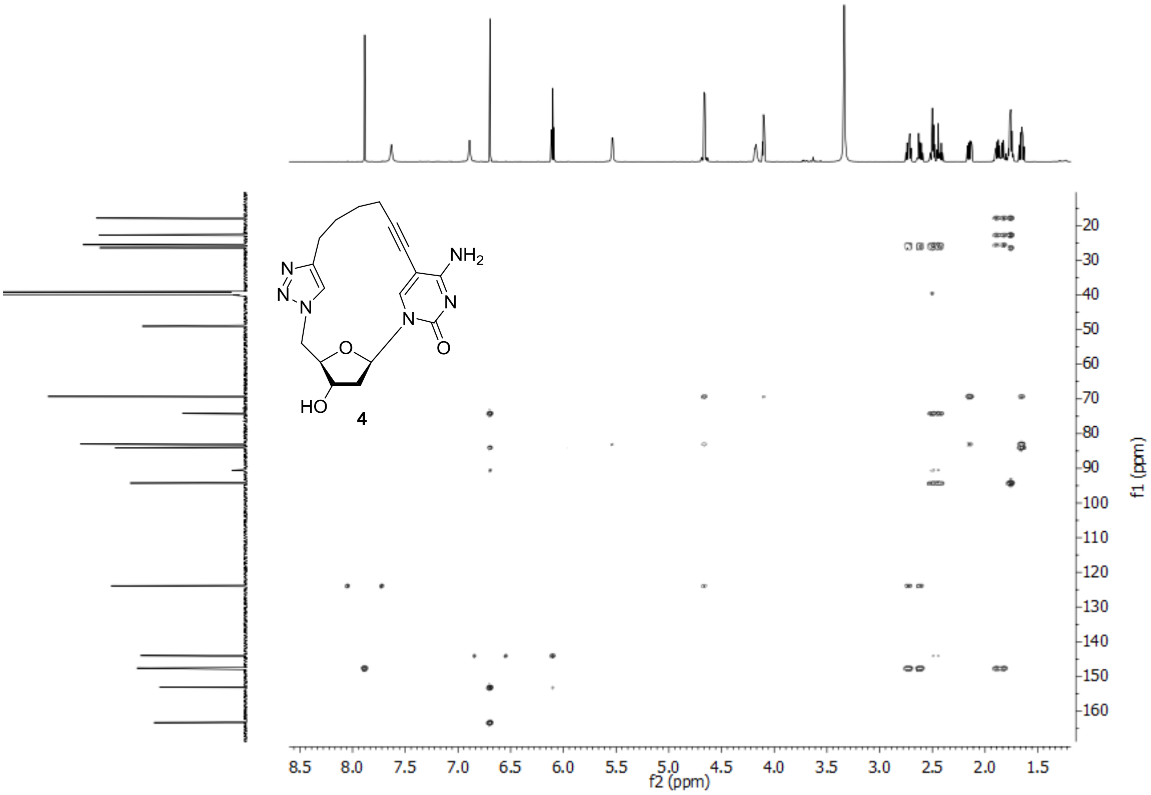

Figure S18. HMBC spectrum of compound **4**.

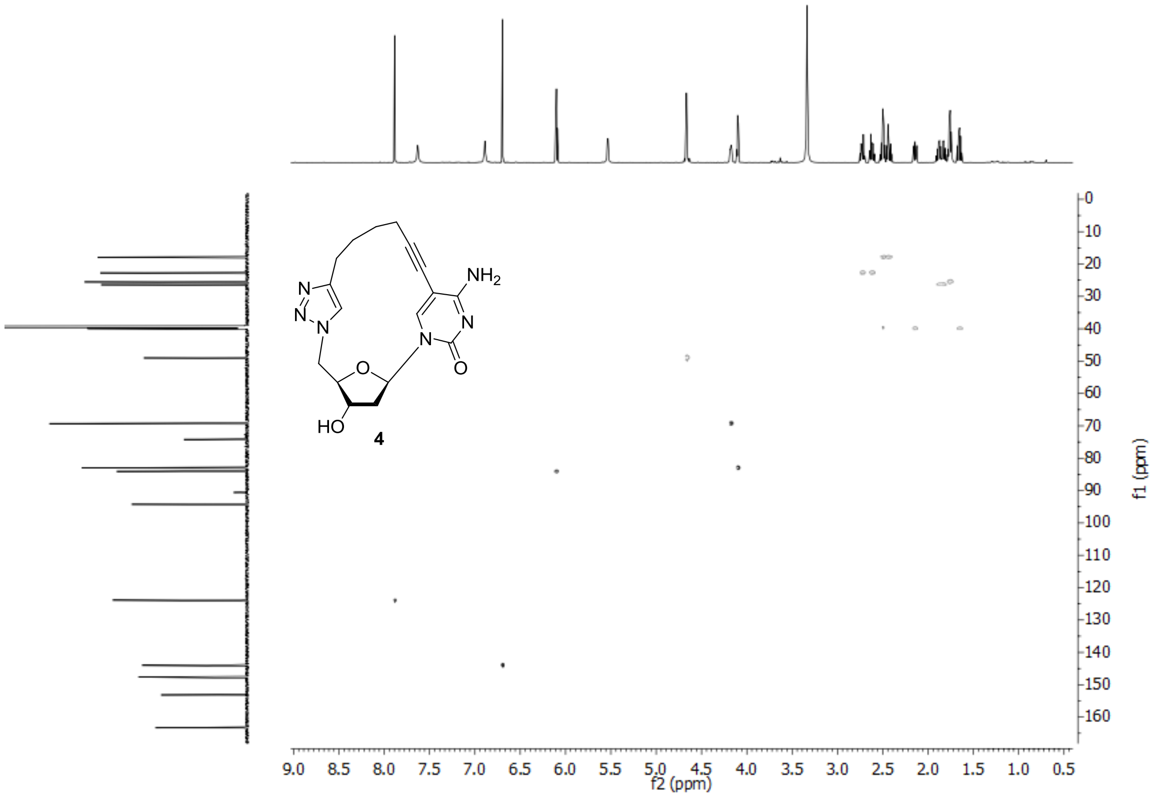

Figure S19. HSQC spectrum of compound **4**.

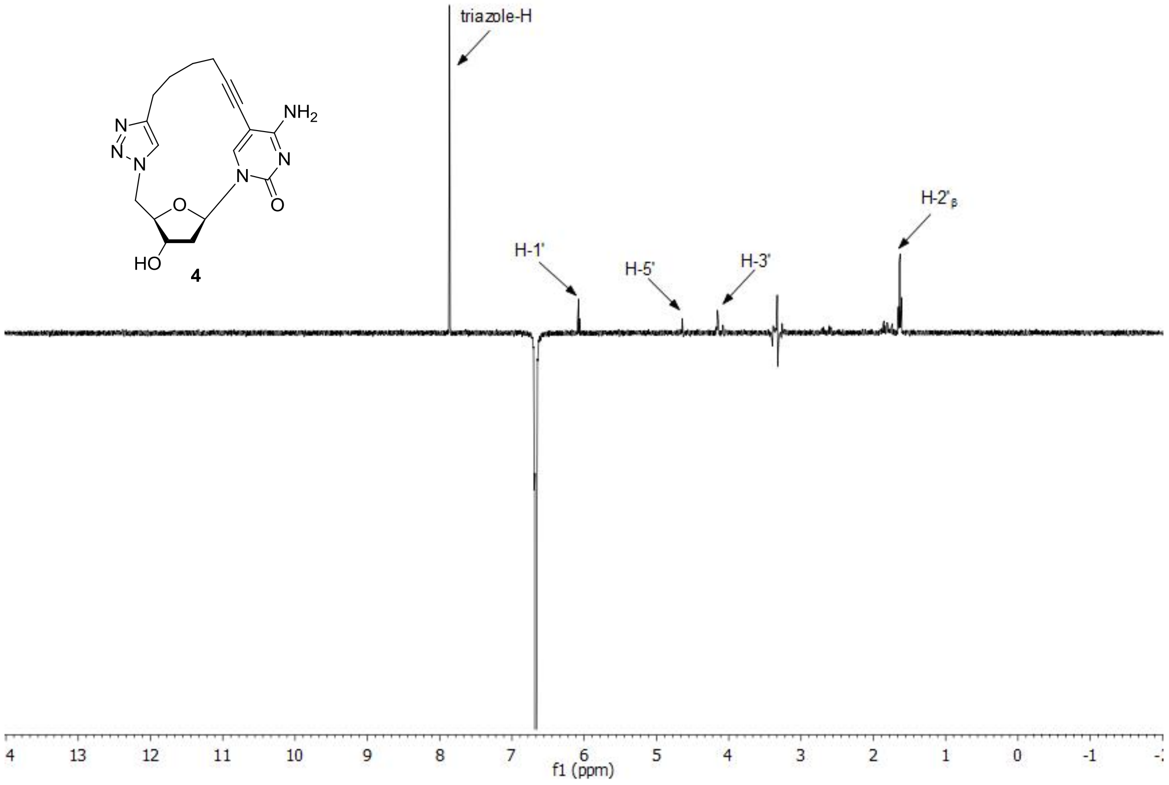

Figure S20. NOE spectrum of compound **4** irradiation of H-6.

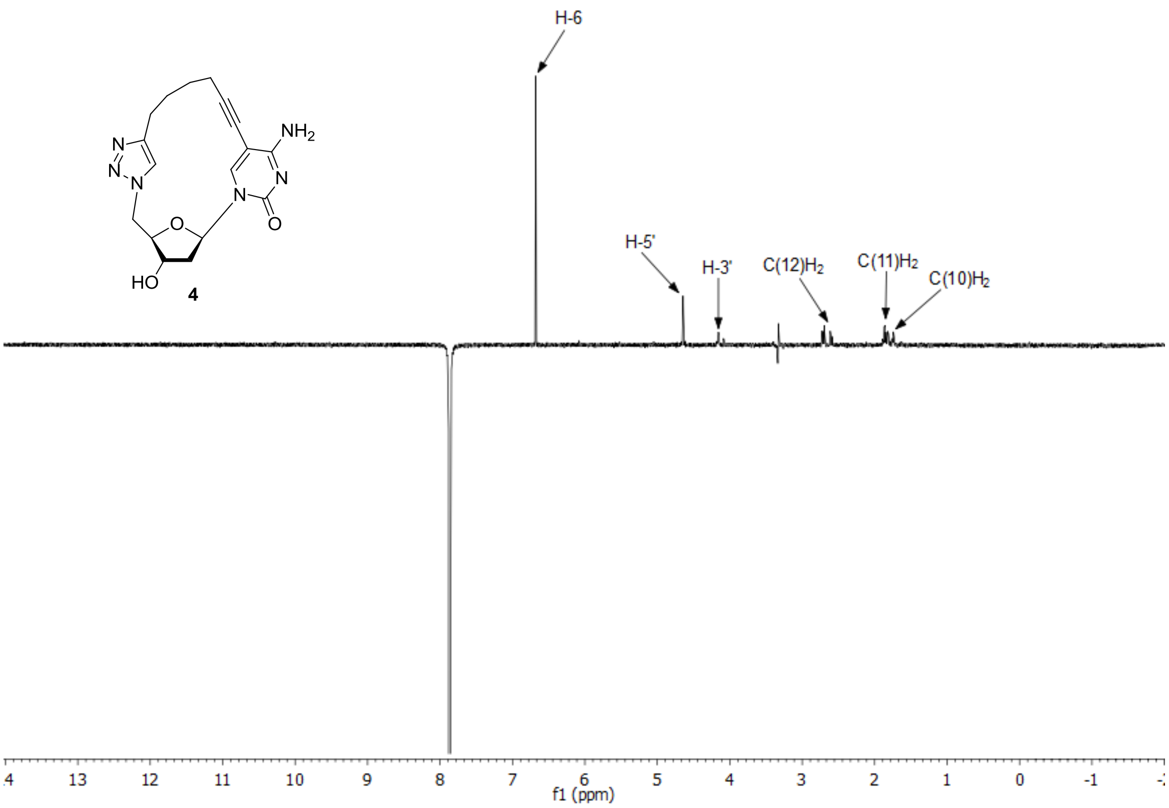

Figure S21. NOE spectrum of compound **4** irradiation of triazole-H.

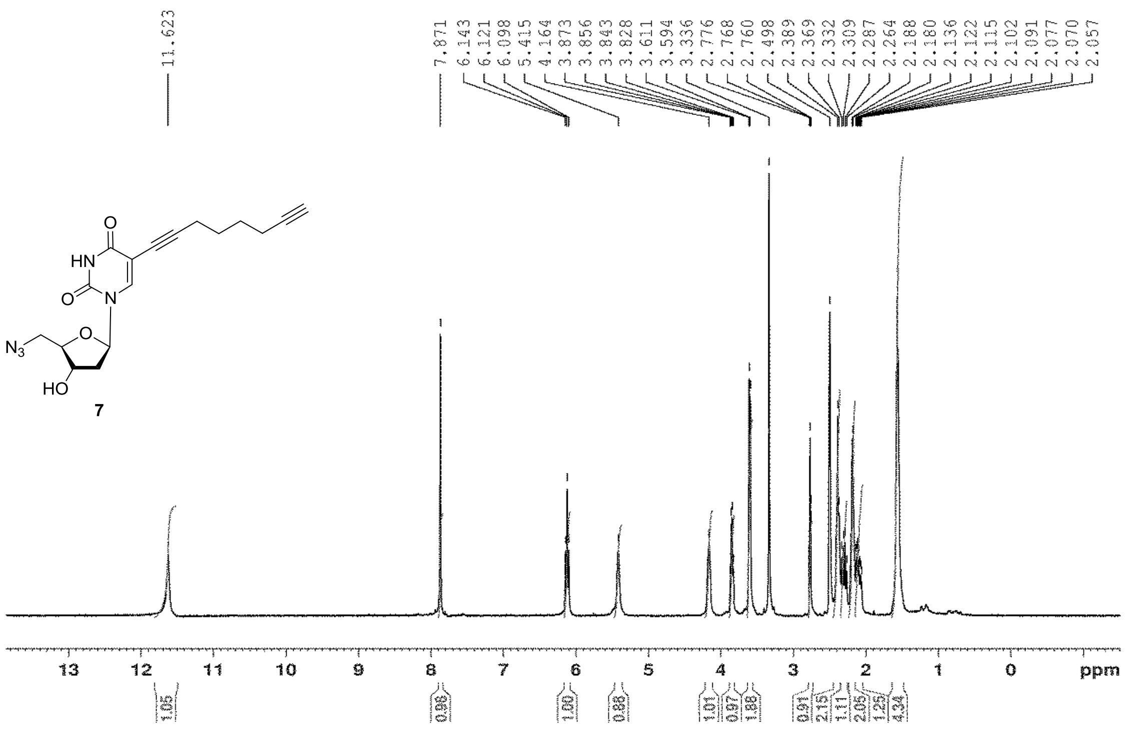

Figure S22. ^{1}H NMR spectrum of compound **7**.

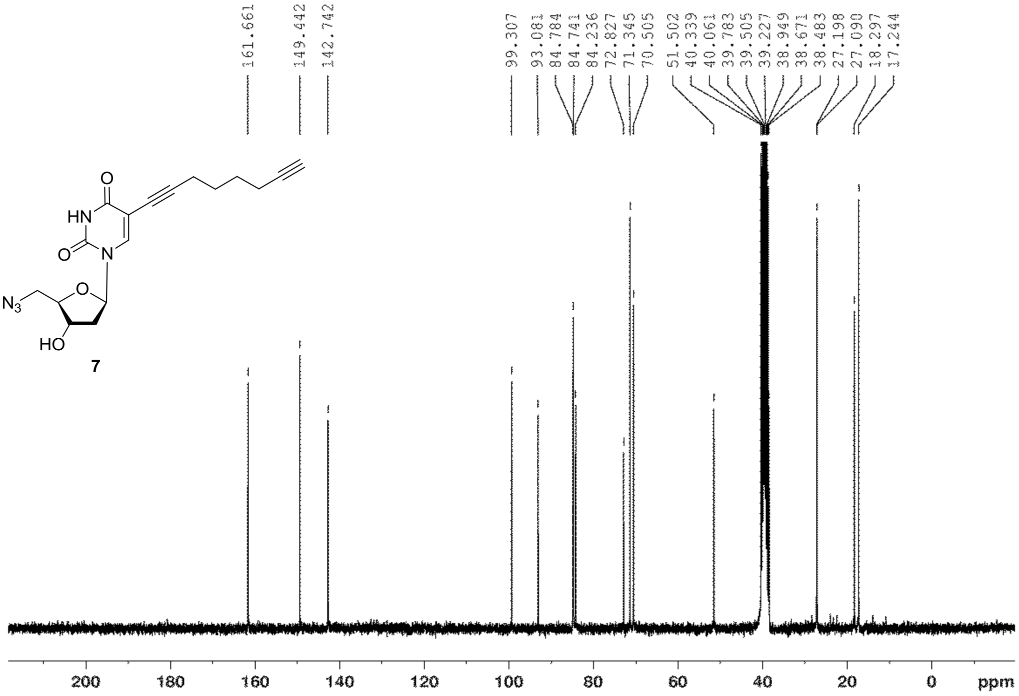

Figure S23. ^{13}C NMR spectrum of compound **7**.

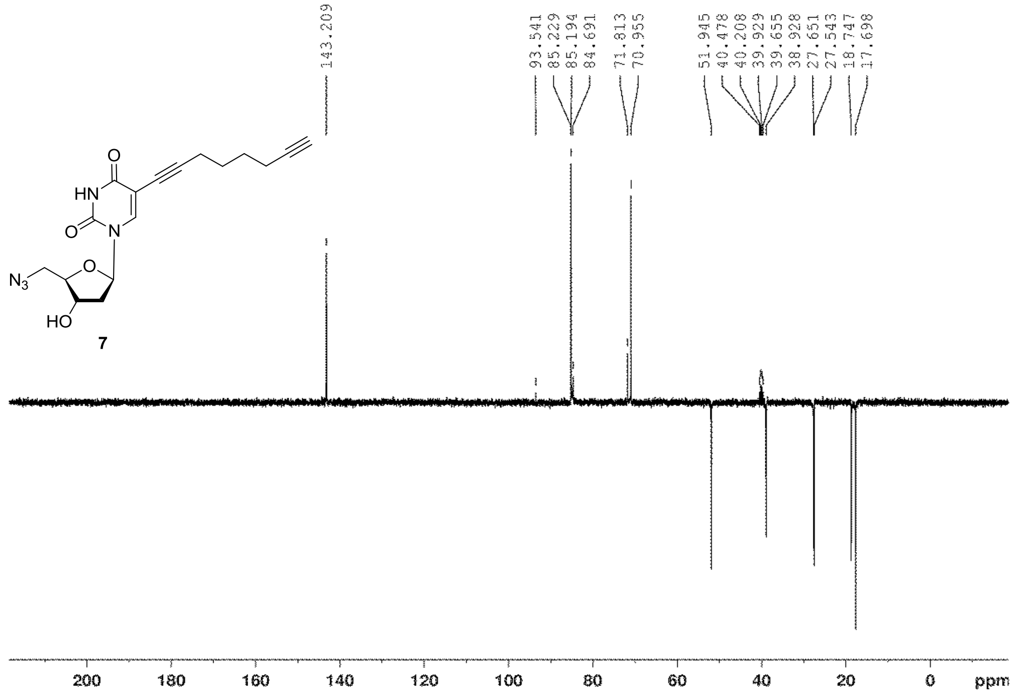

Figure S24. Dept 135 NMR spectrum of compound **7**.

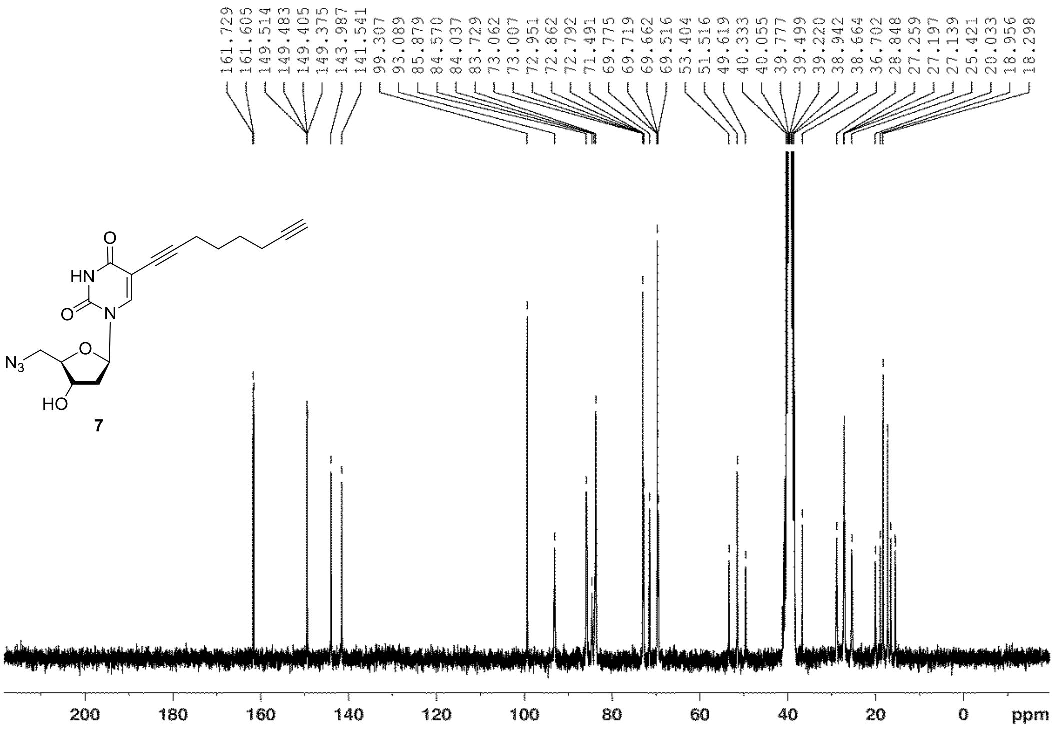

Figure S25. ^{1}H-^{13}C gated-decoupled spectrum of compound **7**.

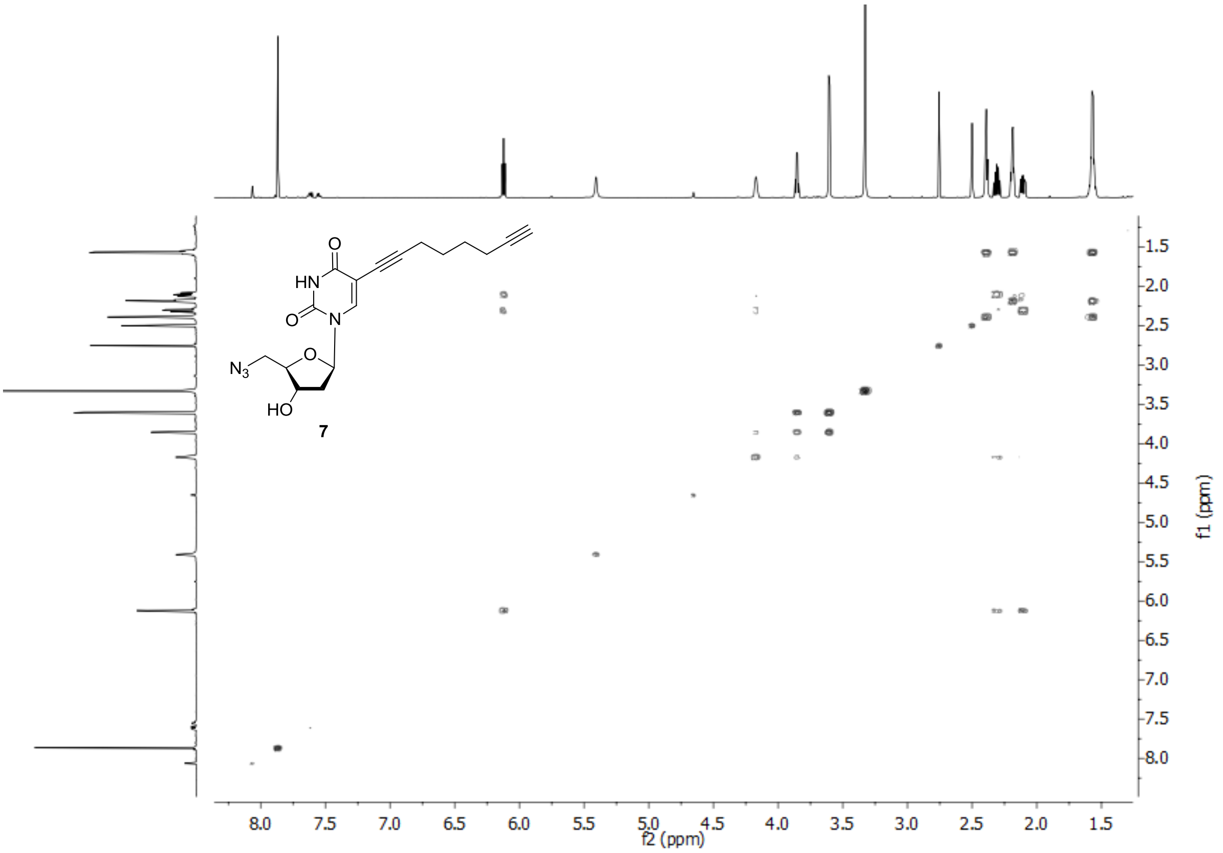

Figure S26. COSY spectrum of compound **7**.

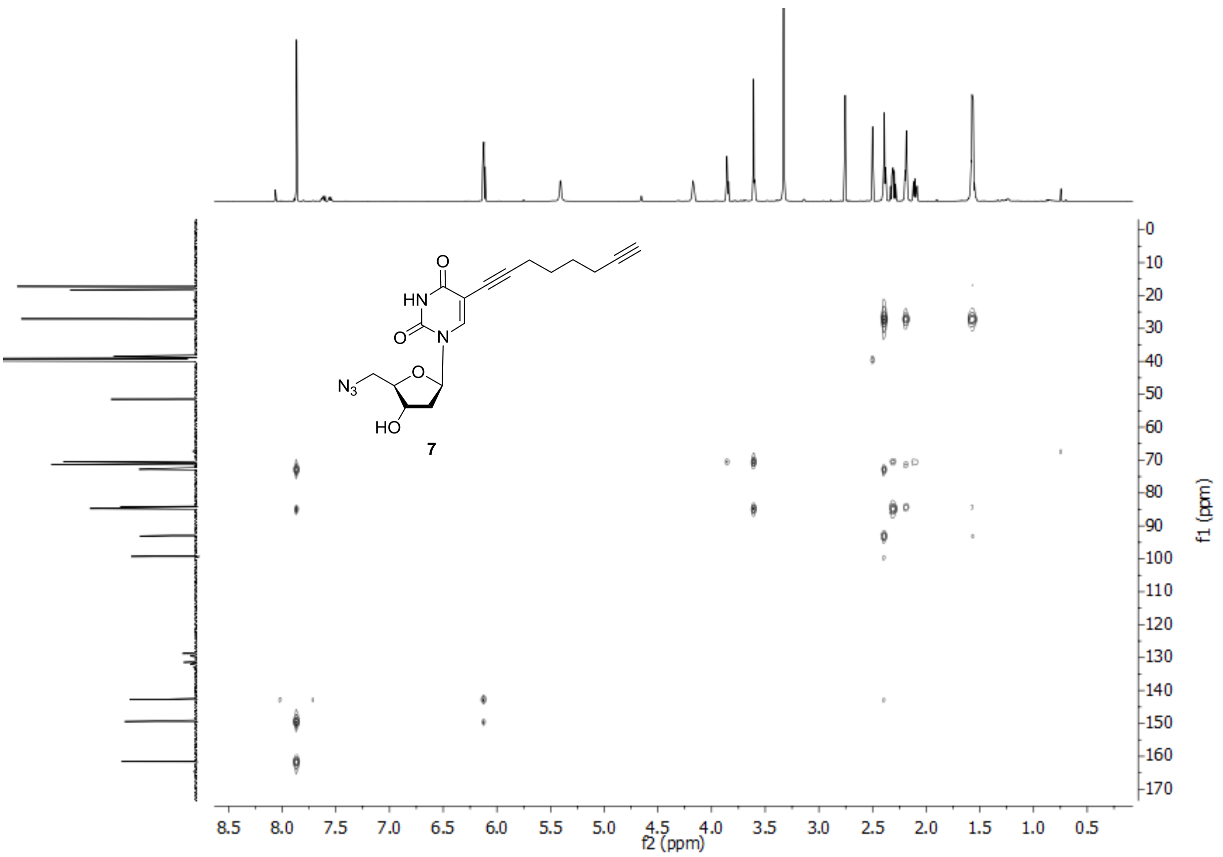

Figure S27. HMBC spectrum of compound **7**.

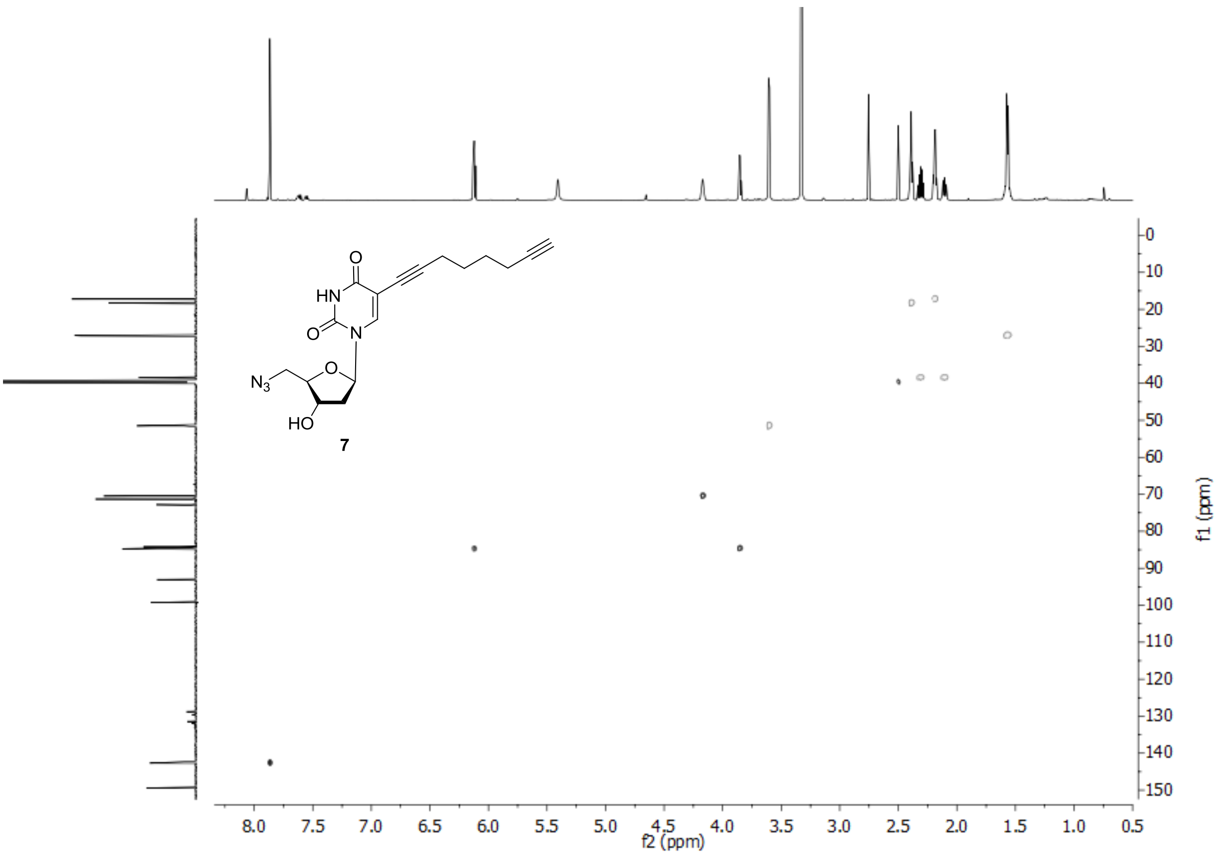

Figure S28. HSQC spectrum of compound **7**.

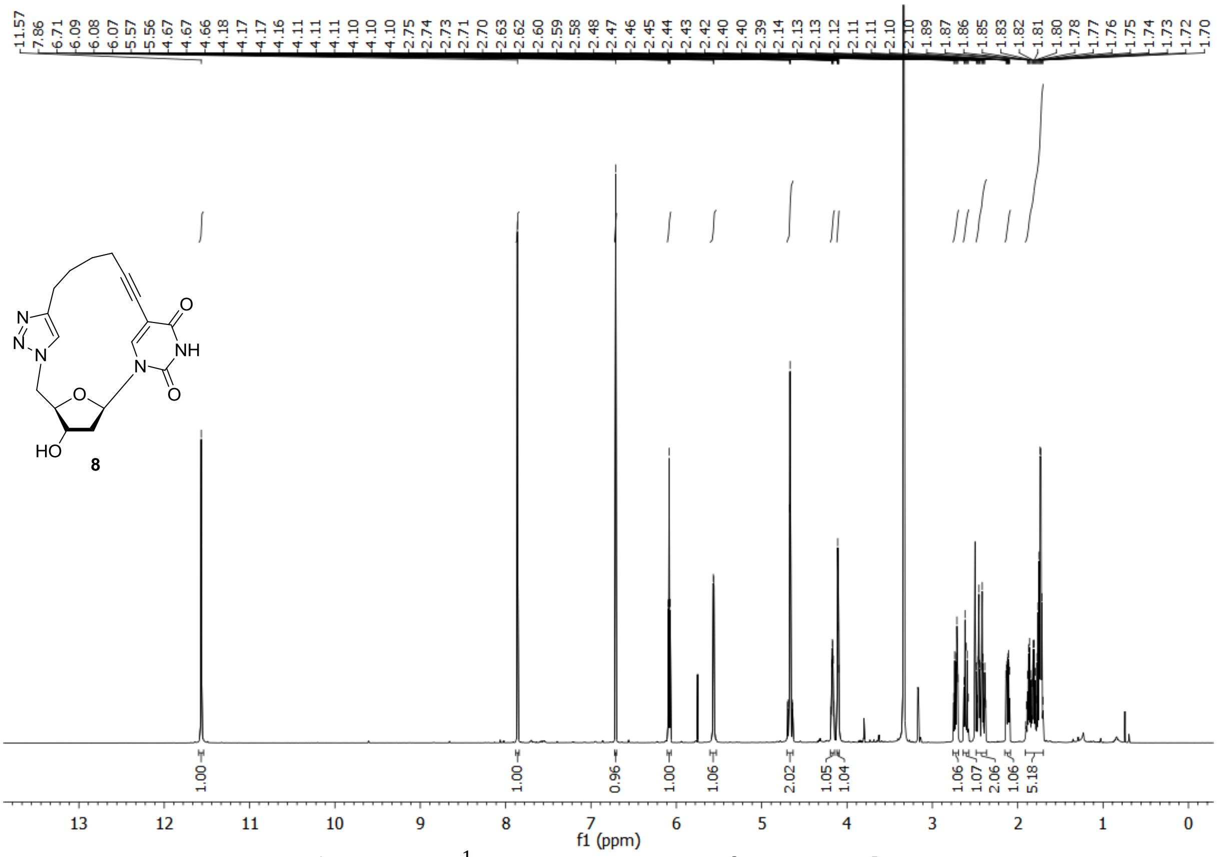

Figure S29. ^{1}H NMR spectrum of compound **8**.

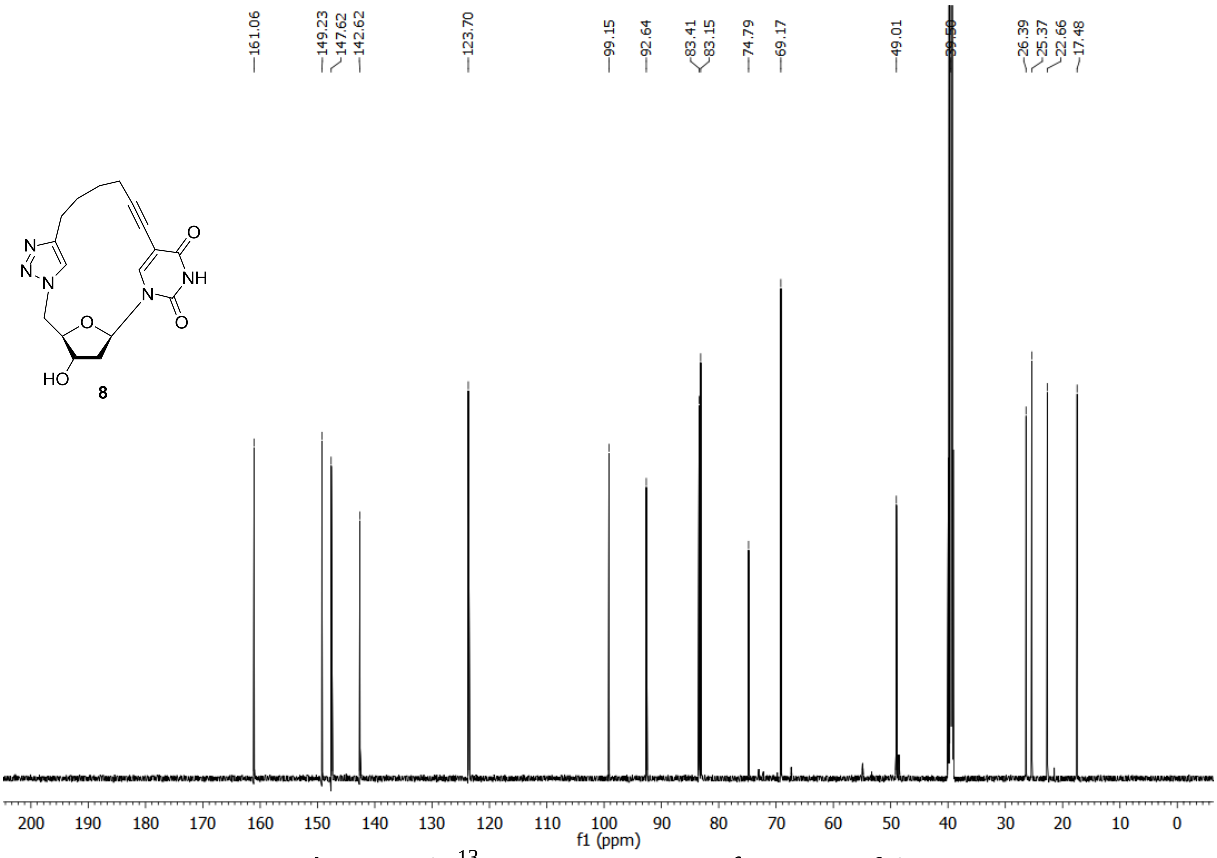

Figure S30. ^{13}C NMR spectrum of compound **8**.

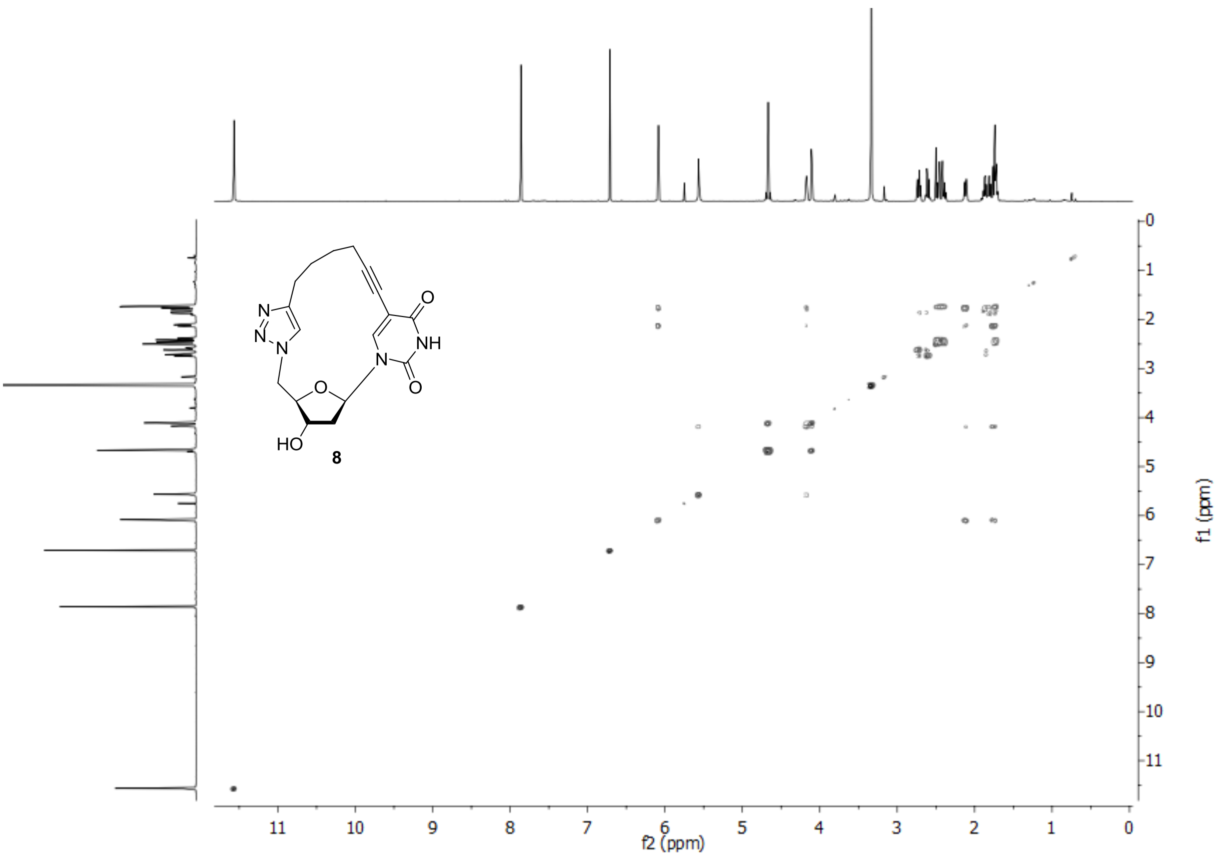

Figure S31. COSY spectrum of compound **8**.

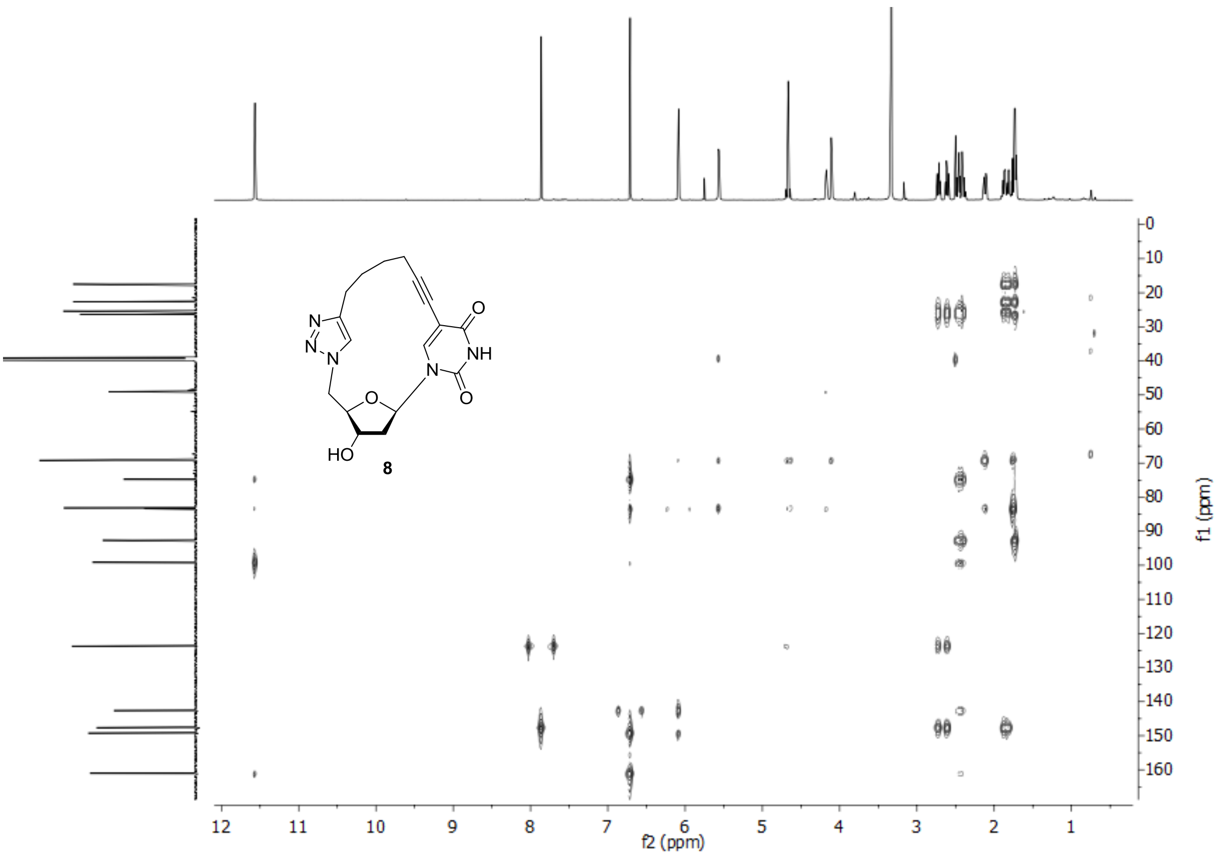

Figure S32. HMBC spectrum of compound **8**.

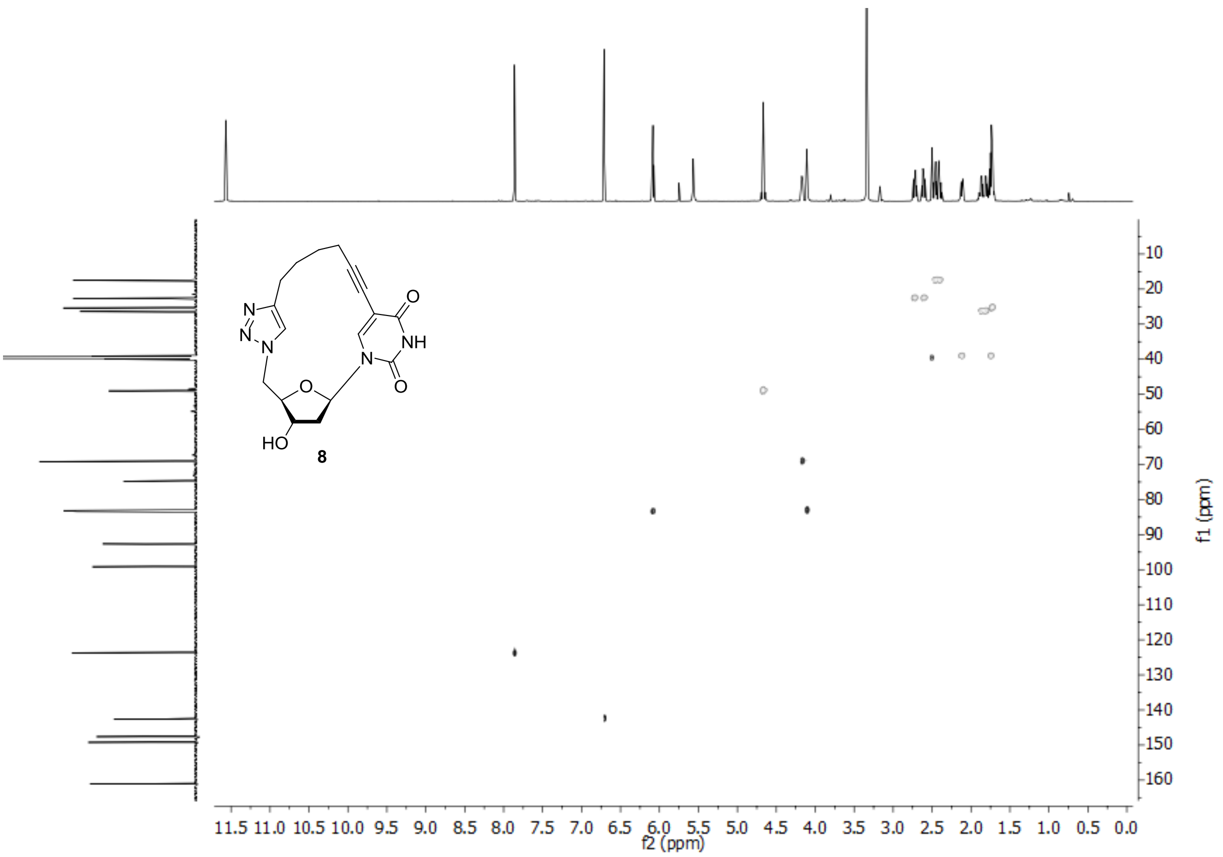

Figure S33. HSQC spectrum of compound **8**.

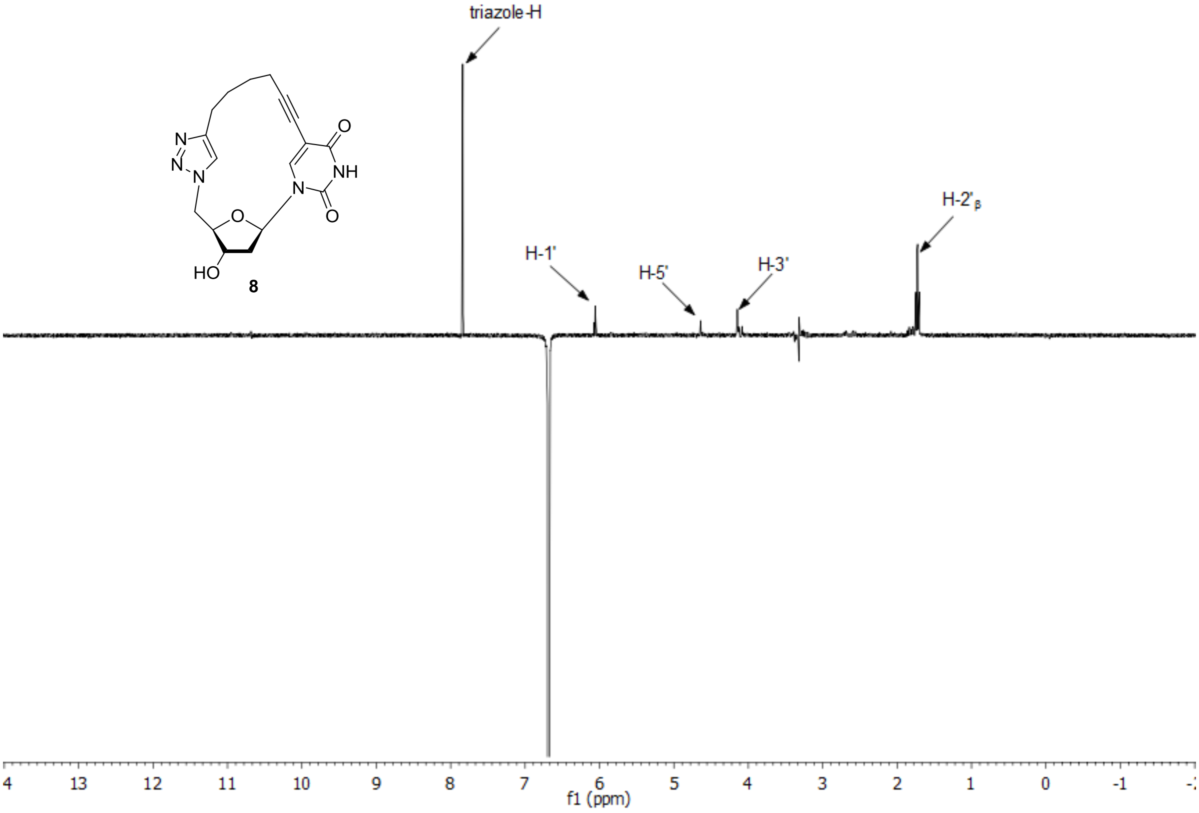

Figure S34. NOE spectrum of compound **8** irradiation of H-6.

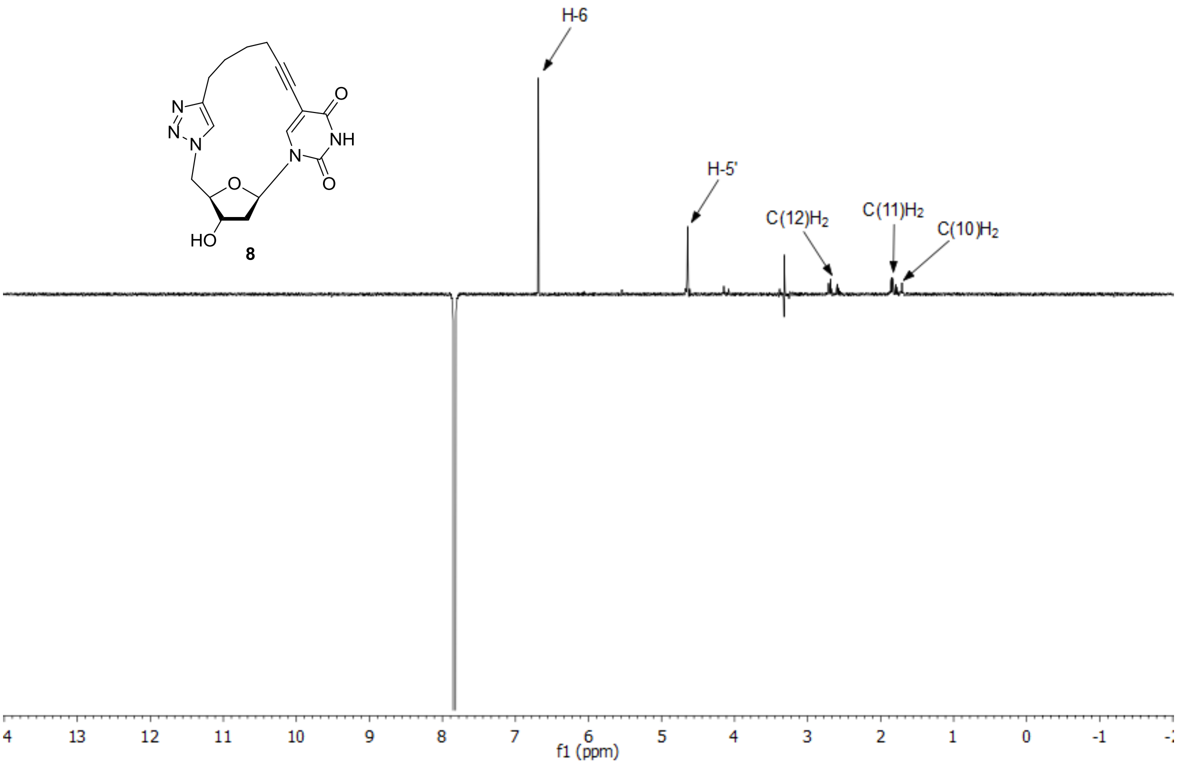

Figure S35. NOE spectrum of compound **8** irradiation of triazole-H.
